# Light and heat control over secondary structure and amyloid-like fiber formation in an overcrowded-alkene-modified Trp zipper†
†Electronic supplementary information (ESI) available: Synthesis of compounds **1–8** and NMR spectra. Isomerization cycle of compound **5** (Fig. S3–S7). Isomerization cycle of compound **1** (Fig. S8–S12). Temperature dependency of CD spectra for compound **1** and **8** (Fig. S13 and S14). 2D-NMR studies on compound **1** (Fig. S15) and 2D-NMR spectra. TEM measurements for compound **1** (Fig. S16 and S17). Nile red experiment (Fig. S18). See DOI: 10.1039/c5sc02735g


**DOI:** 10.1039/c5sc02735g

**Published:** 2015-09-23

**Authors:** Claudia Poloni, Marc C. A. Stuart, Pieter van der Meulen, Wiktor Szymanski, Ben L. Feringa

**Affiliations:** a Centre for Systems Chemistry , Stratingh Institute for Chemistry , Faculty of Mathematics and Natural Sciences , University of Groningen , Nijenborgh 4 , 9747AG Groningen , The Netherlands . Email: b.l.feringa@rug.nl ; Email: w.szymanski@umcg.nl ; Fax: +31-50-3634279; b Department of Radiology , University of Groningen , University Medical Center Groningen , Hanzeplein 1 , 9713 GZ , Groningen , The Netherlands

## Abstract

The external photocontrol over peptide folding, by the incorporation of molecular photoswitches into their structure, provides a powerful tool to study biological processes. However, it is limited so far to switches that exhibit only a rather limited geometrical change upon photoisomerization and that show thermal instability of the photoisomer. Here we describe the use of an overcrowded alkene photoswitch to control a model β-hairpin peptide. This photoresponsive unit undergoes a large conformational change and has two thermally stable isomers which has major influence on the secondary structure and the aggregation of the peptide, permitting the phototriggered formation of amyloid-like fibrils.

## Abstract 2

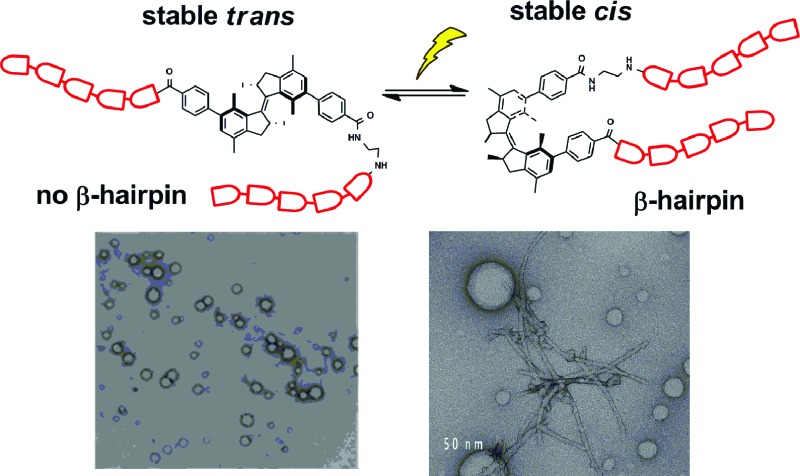
The use of an overcrowded alkene photoswitch to control a model β-hairpin peptide is described. The light-induced, large conformational change has major influence on the secondary structure and the aggregation of the peptide, permitting the triggered formation of amyloid-like fibrils.

## Introduction

Peptides that can assemble into fibers have been intensively studied because they can form hydrogels and they are the precursors of cytotoxic fibers, for example in Alzheimer and Parkinson diseases.^1–3^ Research on these medicinally-relevant peptides aims at understanding the process and the mechanism behind the fibril formation. β-Hairpin peptides are involved in the processes of formation of amyloidogenic fibrils and the proper design of their sequence can lead to the development of inhibitors for amyloidogenesis.^4^

Recently, considerable effort has been devoted to the synthesis of responsive β-hairpins, which can be controlled with external stimuli, such as pH,^5^ temperature^6^ and ionic strength,^7^ among others. Of special interest are systems that use light to modulate peptide folding in order to control biological function.^8,9^ As opposed to the above-mentioned stimuli, light can be delivered with very high spatial and temporal precision and control over the intensity and wavelength of irradiation. The creation of light-responsive peptide hybrids relies on covalent introduction of molecular photoswitches.^8,9^ Several photoresponsive β-hairpins were synthesized by incorporating either azobenzene,^10–13^ stilbene,^14^ or hemithioindigo^15^ photochromic units into the putative turn-region of the β-hairpin structure. These powerful tools have already shown their potential in delivering insights into the mechanism and kinetics of β-hairpin formation and, through that, information on the process behind the formation of cytotoxic fibrils involved in different diseases.^11,16,17^

Photoswitchable β-hairpins are also considered for their ability to form hydrogels: they can be useful in tissue engineering, drug delivery and biosensing.^10^ Simple β-hairpins, bearing an azobenzene unit, have been used to modulate viscoelasticity of a peptide hydrogel.^10^ However, the small number and insufficient structural diversity of molecular photoswitches that have been evaluated for their use in controlling peptide conformation limits the scope of potential photoregulated systems. Furthermore, in all cases of switches used, only two photoisomeric states can be addressed, one of which is usually thermally unstable.

**Fig. 1:**
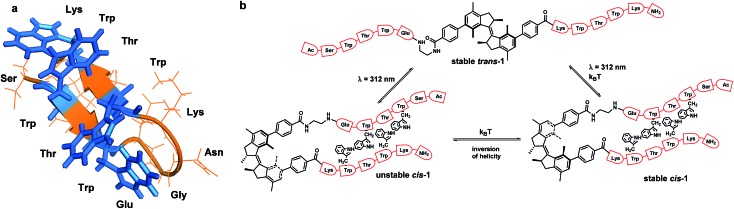
Trpzip modified with overcrowded alkene. (a) Trpzip reported by Cochran *et al.* ^22^ (b) Rotary cycle for β-hairpin bearing overcrowded alkene. Picture created from PDB ; 1LE0 (ref. 22).

Here we report, for the first time, the application of an overcrowded alkene-based switch^18^ for the control of a β-hairpin peptide structure (Fig. 1b) and show it to be effective in the control of both peptide conformation and aggregation. This overcrowded alkene switch has two stable stereoisomers, *trans* and *cis* form, which strongly differ in shape and helicity. The conversion between these two states occurs *via* an intermediate, a metastable stereoisomer (unstable-*cis*) with a half-life in the order of days (Fig. 1b). Irradiation of the *trans* isomer leads to *trans*–*cis* isomerisation at the central olefinic bond and formation of the unstable *cis* isomer.

Thermal relaxation of this form leads to helicity inversion and stable *cis* isomer is obtained. Irradiation of the stable *cis* isomer provides a short-lived unstable *trans* isomer, which, on the scale of seconds at room temperature undergoes transformation into the stable *trans* form, thereby completing a switching cycle of the overcrowded alkene switch.

We expected that this photoresponsive molecule, upon introduction into a peptide backbone, would provide more information on the mechanism and dynamic of the formation of secondary structure, as compared to conventional photoswitches; for example, the metastable stereoisomer could offer the possibility to reveal the presence of intermediates of this process in a spatially and temporally controlled manner. Of importance is also the presence of more than one thermally stable form that can be accessed with light, which could permit to follow the processes behind the peptide folding without them being affected by the thermal reisomerisation, as is often the case for azobenzenes and hemithioindigos. The photostationary state for the overcrowded alkene switch, used here, is higher than the one observed for many other photoswitches.^9^ Moreover, the photoisomerization between the stable states imposes a different geometrical change with respect to azobenzenes or other switches used so far. The overcrowded alkene switch was already successfully used for tuning the enantioselectivity of reactions,^18,19^ photocontrolling magnetic interactions^20^ and ion binding.^21^ Here, we show that an overcrowded alkene switch can be used to control peptide conformation and aggregation.

The β-hairpin sequence (Fig. 1a), used in our design, is called trpzip and it was introduced by Cochran, Starovasnik and co-workers.^22^ The trpzips are stable β-hairpins and they fold in a monomeric form without requiring metal binding.^22^ They are stabilized by cross-strand pairs of indole rings. The turn region can vary in composition: the pair NG favours a turn I′ while GN or ^D^PN favour turn II′ (Fig. 1a).^22^ In our approach, the overcrowded alkene switch was inserted in the turn region and we analysed the effect of photoisomerization on the secondary structure. We envisioned that the *trans* photoisomer of the overcrowded alkene switch does not mimic the turn and therefore it wouldn't form any defined structures, while the stable and unstable *cis* stereoisomer, by bringing the two strands in close proximity, would promote β-hairpin structure formation. This would permit photo-triggered formation of secondary structure of a β-hairpin peptide. Notably, trpzip peptide was already modified by Moroder^23^ with an azobenzene switch at the same position, which allows to compare the effects of the overcrowded alkene and the azobenzene switches.

## Results and discussion

### Design and synthesis

**Scheme 1:**
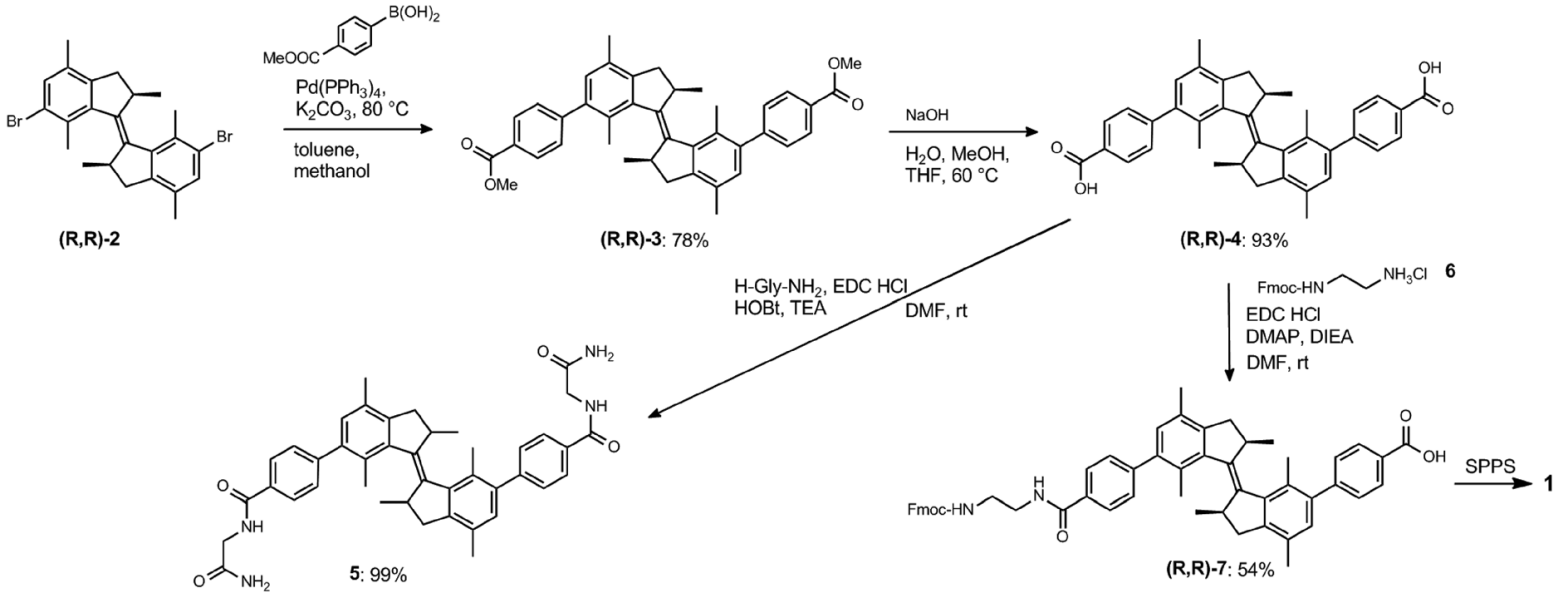
Synthesis of compound **7**, a building block for SPPS bearing an overcrowded alkene, and model compound **5**.

For the convenient and generally-applicable preparation of peptides with overcrowded alkene introduced into the peptide backbone, we designed a switch-bearing building block **7** (Scheme 1) that can be applied in standard Fmoc-based solid phase peptide synthesis (SPPS). Compound **7** was synthesized from previously-reported enantiopure precursor **2**.^24^ Suzuki–Miyaura coupling of compound **2** with 4-methoxycarbonylphenyl boronic acid, followed by ester hydrolysis, yielded the dicarboxylic acid **4**. Subsequent mono-amide formation with Fmoc-ethylene diamine **6** lead to the desired product **7** (Scheme 1). The ethylene diamine linker provides the amine functionality. It might, on the other hand, bring undesirable conformational flexibility to the system. However, Gogoll *et al.* show that this flexibility is not a limiting factor for the controlled folding process.^25^

**Fig. 2:**
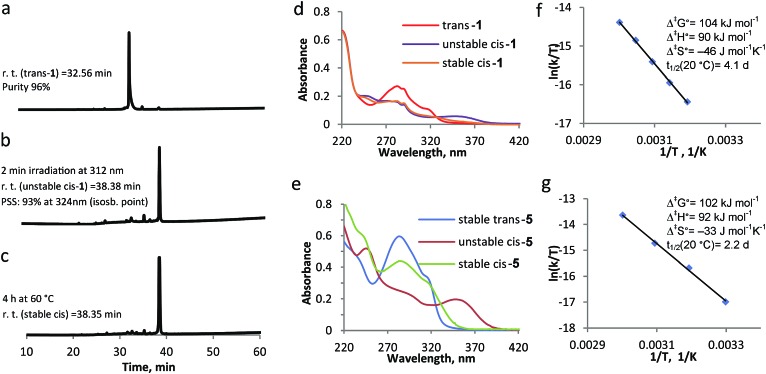
Switching cycle for modified β-hairpin **1** and model compound **5** followed by UV-vis spectroscopy and HPLC. (a) HPLC trace for *trans*-**1**. (b) HPLC trace after 2 min irradiation at *λ* = 312 nm to form unstable *cis*-**1**. (c) HPLC trace after 4 h at 60 °C to form stable *cis*-**1**. (d) UV-vis spectra of *trans*-**1**, unstable *cis*-**1** and stable *cis*-**1**. (e) UV-vis spectra of *trans*-**5**, unstable *cis*-**5** and stable *cis*-**5**. (f) Eyring plots obtained by following the decrease in absorbance at 350 nm at different temperatures, with the associated activation parameters for the transition unstable *cis*–stable *cis*, for compound **1**. (g) Eyring plots obtained by following the decrease in absorbance at 350 nm at different temperatures, with the associated activation parameters for the transition unstable *cis*–stable *cis*, for compound **5**.

The β-hairpin-switch hybrid **1** (Fig. 1b) was synthesised by a standard protocol for Fmoc-based SPPS, using building block **7**. However, the removal of protecting groups (Boc, Trt, O*t*Bu, Pbf) and cleavage from the resin, using standard cleavage cocktails that contain 95% trifluoroacetic acid, did not lead to the isolation of the desired product. This could be explained by the acid-sensitivity of the highly constrained double bond of the overcrowded alkene. The use of a highly acid-sensitive Sieber amide resin, together with acid-sensitive protecting groups for the side-chains, like Trt and O-2-PhiPr, permitted the use of a cleavage cocktail with <50% of TFA and led to the isolation of the desired bis-peptide-overcrowded alkene product (see ESI† for details of procedures). The product *trans*-**1** was purified by semi-preparative HPLC and lyophilized. MALDI-TOF confirmed the product and the purity was found by HPLC to be 96% (Fig. 2a, Fig. S1, ESI†).

### Switching cycle of overcrowded alkene-bis-peptide

UV-vis and NMR spectroscopies are generally used to study the switching cycle of overcrowded alkenes.^18,20^ The isomerisation around the double bond is induced by light and heat and it is accompanied by inversion of helicity (Fig. 1b). Irradiation of *trans*-**1** isomer at *λ* = 312 nm leads to the unstable *cis*-**1** isomer, which is characterised by the appearance of a band at 350 nm in the UV-vis spectrum. Unstable *cis* isomer converts to stable *cis* form in a thermal isomerization process, which is accompanied by the disappearance of the 350 nm absorption band. If irradiation at *λ* = 312 nm is applied to the stable *cis* isomer at low temperature, the unstable *trans* stereoisomer is presumably formed, which quickly undergoes relaxation to the stable *trans* from.^18,19,21,26^

To study the influence that the modification of an overcrowded alkene with a β-hairpin peptide has on its switching cycle, we synthesized and analysed reference compound **5** (Scheme 1). This molecule is a similar overcrowded alkene switch functionalized with two glycinamides *via* amide bonds, representing the simplest peptide-bearing overcrowded alkene switch. We compared the kinetics of the switching cycle of compounds **1** and **5** and analyzed them both in the entropic and enthalpic terms (Fig. 2), reasoning that the differences in the enthalpic term would imply interactions between the two strands in compound **1**.

On the other hand, the possible difference in the entropic term could be attributed to the increased length of the peptidic side chains in **1**, compared to **5**, since the rotation in **1** would require more pronounced reorganization of solvent molecules.^27^ The switching cycle of **1** was studied in methanol, using HPLC as an additional analytical method, besides CD, UV-vis spectroscopy and NMR. The irradiation of *trans*-**1** at *λ* = 312 nm for 2–15 min leads to unstable *cis*-**1**: the photostationary state (PSS) was determined by HPLC to have a 93 : 7 unstable *cis* : *trans* ratio (Fig. 2b). Subsequently, the thermal isomerisation, from unstable *cis*-**1** to stable *cis*-**1**, was achieved by warming the sample up at 40–50 °C for 4–6 h. While HPLC analysis shows that there is no significant difference in polarity between unstable *cis*-**1** and stable *cis*-**1** (Fig. 2b and c), this transformation is apparent from the change in the UV-vis spectrum (Fig. 2d) and NMR spectrum (Fig. 5b, see below for discussion). The decrease in absorbance at *λ* = 350 nm, corresponding to the conversion of unstable *cis*-**1** to the stable form, was followed in time at different temperatures and the thermodynamic parameters were calculated using the Eyring equation (Fig. 2f). The established Gibbs free energy of activation (Δ^‡^ *G*°) was 104 kJ mol^–1^, the enthalpy of activation (Δ^‡^ *H*°) was 90 kJ mol^–1^ and the entropy (Δ^‡^ *S*°) at rt was –46 J mol^–1^ K^–1^. The half-life of unstable *cis*-**1** is 4.1 d at rt. A similar study was performed on model compound **5**, giving the Δ^‡^ *G*° of 102 kJ mol^–1^ and the half-life of 2.2 d at rt (Fig. 2g). The difference in Δ^‡^ *G*° for the thermal helix inversion between compounds **1** and **5** is mainly due to the entropic term (Fig. 2f and g). Therefore we attribute the difference in kinetics not to the formation of secondary structure but to the mobility of the two arms connected to the switch, either due to aggregation or to the length of the these two arms as reported for an overcrowded alkene modified with different arms acting as a molecular stirrer.^27^ In fact, the isomerisation is affected by the length of the rigid substituents in viscous solvents and the differences in kinetics are dominated by entropy effects.^27^

### Circular dichroism

Circular dichroism (CD) spectroscopy provides additional information about the structure of the peptide; edge-to-face interaction of the indole side chains of the tryptophan moieties has been reported as an indication of β-hairpin formation in trpzip peptides.^13,22^ The CD spectrum for the β-hairpin shows an exciton coupling at *λ* = ∼220 nm.^28^ When indole groups of the tryptophans interact with each other, typically an increase of molar ellipticity of this band is observed.^22^ Temperature influences the stacking of tryptophans and, therefore, it is possible to determine the temperature of denaturation of the β-hairpin, following the decrease of the molar ellipticity of the band at *λ* = 220 nm as a function of temperature.^22^

**Fig. 3:**
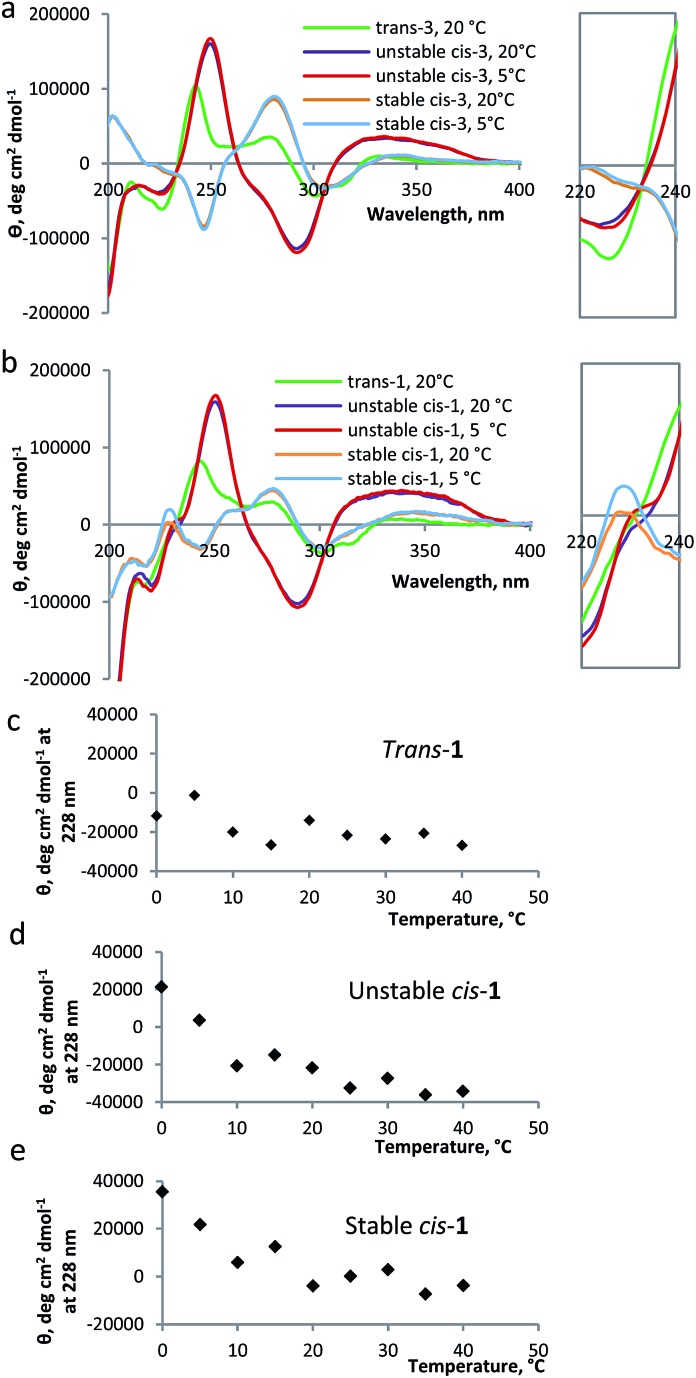
CD spectra for modified β-hairpin **1** and compound **3**. (a) CD spectra of *trans*-**3** at 20 °C, unstable *cis*-**3** at 20 °C and 5 °C, stable *cis*-**3** at 20 °C and 5 °C (24 μM in methanol). Inset: zoom in on the corresponding band at 230 nm. (b) CD spectra of *trans*-**1** at 20 °C, unstable *cis*-**1** at 20 °C and 5 °C, stable *cis*-**1** at 20 °C and 5 °C (24 μM in methanol). Inset: zoom in on the corresponding band at 230 nm. (c) Molar ellipticity (*θ*) at 228 nm for *trans*-**1** at different temperatures. (d) Molar ellipticity (*θ*) at 228 nm for unstable *cis*-**1** at different temperatures. (e) Molar ellipticity (*θ*) at 228 nm for stable *cis*-**1** at different temperatures.

The CD spectrum of compound **1** has two components: the CD signals due to the overcrowded alkene chromophore and those resulting from the tryptophan indole groups. To estimate the first of these components, a simple switch compound **3** (Scheme 1) was analyzed (Fig. 3a). The switch shows a strong CD signal in the range of 200–400 nm: the CD spectrum of compound **3** in the *trans* form shows two positive signals at 243 nm and at 285 nm; the unstable *cis* form shows one positive signal at 253 nm and one negative signal at 295 nm, while the stable *cis* has a negative signal at 249 nm and a positive one at 286 nm, comparable to results reported previously for other overcrowded alkenes.^18^ The helical inversion during the isomerization of unstable *cis* to stable *cis* is evident from the change in sign for the bands at ∼250 nm and at ∼290 nm (Fig. 3a).

As compared to the results obtained for compound **3**, the CD spectra of compound **1**, in unstable and stable *cis* forms, show an additional band at 228 nm, which is characteristic for the tryptophan moiety (Fig. 3b).^22^ For unstable *cis*-**1** and stable *cis*-**1**, the molar ellipticity of the band at 228 nm increases when the temperature is decreased, while for the *trans* form it remains largely unchanged (Fig. 3c, d and e). This temperature-dependence indicates that, especially at low temperature, unstable *cis*-**1** and stable *cis*-**1** can form the β-hairpin structure, while *trans*-**1** does not. It is not possible to precisely quantify the content of β-hairpin, because the CD absorption band at 228 nm has a strong contribution of the switch unit, especially for unstable *cis*-**1**. For stable *cis*-**1**, the content in secondary structure at 0 °C can be estimated to be ∼50% compared to the natural β-hairpin (by comparison of Fig. S13 and S14, ESI†).

### NMR study

**Fig. 4:**
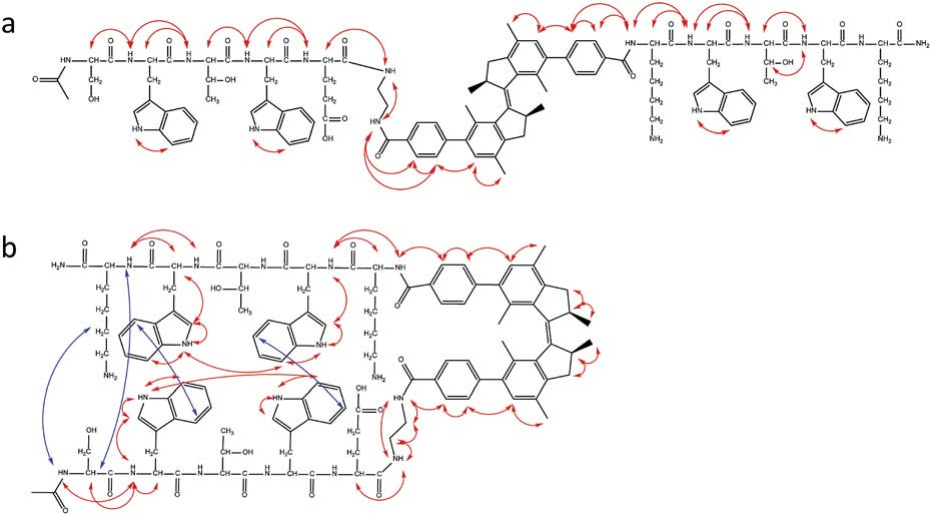
Cross peaks scheme obtained from NOESY NMR spectra. (a) NOE signals for *trans*-**1** (2.9 mM in methanol-d_3_) at rt. (b) NOE signals for *cis*-**1** (2.9 mM in methanol-d_3_) at 5 °C. Red lines indicate intra-strand cross peaks and blue lines indicate inter-strand cross peaks. See ESI† for the spectra.

**Fig. 5:**
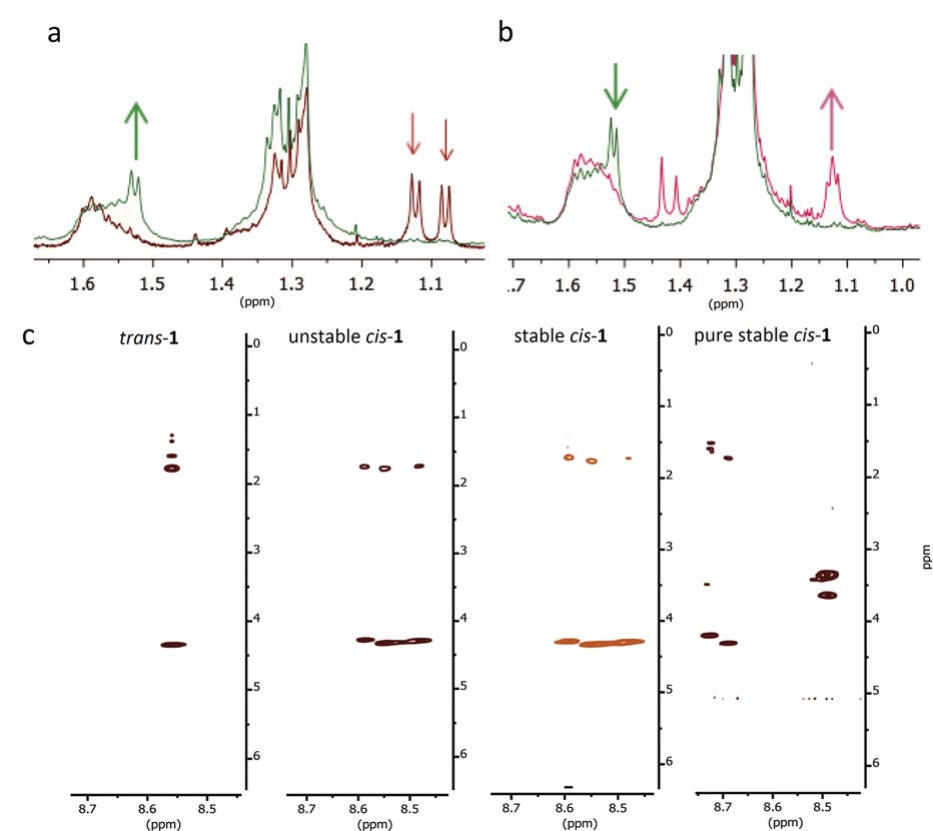
Switching cycle for the modified β-hairpin **1** followed by NMR spectroscopy. (a) Characteristic region for the CH_3_ signals before irradiation (*trans*-**1**; red line) and after irradiation (unstable *cis*-**1**; green line) at 312 nm. (b) Characteristic region for the CH_3_ signals before (unstable *cis*-**1**; green line) and after warming up at 50 °C for 4 h (stable *cis*-**1**; pink line). (c) TOCSY signals (8.5–8.7 ppm) for Lys-6 for *trans*-**1**, unstable *cis*-**1** and stable *cis*-**1** at rt and the purified stable *cis*-**1** at 5 °C (for full spectra, see ESI†).

With an indication on the formation of β-hairpin from the CD measurements, we further studied the nature of this folded structure by NMR analysis of the peptide hybrid **1** in methanol-d_3_: a solvent, which was also used for the study of β-hairpin peptide modified with azobenzene.^13^ ^1^H-NMR, COSY, TOCSY and NOESY spectra were recorded, a set that provides the possibility to assign the proton signals in proteins or peptides using Wüthrich's method.^29^ The measurements were performed for the three different isomers of peptide-switch hybrid **1**. *trans*-**1** gives very sharp signals in the spectra (see 1D and 2D-NMR studies with compound **1**, ESI†), indicating the presence of monomeric species. All the protons in the backbone and in the side-chains were identified, apart for the indole signals of the tryptophans. NOE signals between amino acids in the two strands of the overcrowded alkene were not found (Fig. 4a). The irradiation at *λ* = 312 nm promotes the formation of unstable *cis*-**1** and the warming up at 50 °C for 4 h leads to the formation of stable *cis*-**1**. These two processes were monitored by following the shift of the signals of the two methyl groups in the allylic position of the overcrowded alkene (Fig. 5a and b). The stereochemistry was assigned based on the similar spectral properties of previously reported analogous compounds.^18,19,21^ In the *trans* form, the chemical shift values corresponding to these protons are 1.08 and 1.13 ppm (Fig. 5a); in the unstable *cis* form, one of these protons is visible at 1.52 ppm (Fig. 5a). Although the PSS ratio is >90%, it was not possible to identify clearly the signals of the peptidic region in the unstable *cis* form. Unfortunately the durations of the NMR experiments are in the same range as the thermal stability of the unstable *cis* isomer, therefore structural information can be obtained only for stable *cis* form. For the stable *cis* form, the protons mentioned above have chemical shift of 1.13 ppm (Fig. 5b). Based on the number of signals for every amino acid (for example Lys-6, Fig. 5c) in the stable *cis* form, it seems that two or more species coexist. Such multiplication of NMR signals has been observed before *e.g.* in the case of peptide GNNQQNY, and was attributed to the formation of aggregates of different architecture.^30^ To exclude any degradation of the *cis* forms that would lead to new species visible in NMR spectra, the stable *cis*-**1** was purified by semi-prep HPLC and again analysed by NMR.

The NMR study for stable *cis*-**1** was conducted at rt and 5 °C (see 2D-NMR studies on compound **1**, ESI†). At rt, the NMR spectra were sharp and well-resolved, and few weak cross peaks between the two strands were observed that became more intense upon decreasing the temperature (Fig. 4b). At 5 °C the peaks became broader and two or more coexistent structures are formed (for example shown for the Lys-6 signals, Fig. 5c). The decreased temperature, probably, provokes aggregation; it was not possible to invert the process by increasing the temperature.

In summary, *trans*-**1** does not seem to adopt any secondary structure and exists in a monomeric state. Unstable and stable *cis*-**1** form aggregates and the decrease in temperature promotes this process. There is more than one pattern of signals for some amino acids (Lys-6, Thr-3, Thr-8) and this behaviour is attributed to the coexistence of more than one aggregate.^30^

### TEM and cryo-TEM

To identify the structure formed upon aggregation of compound **1**, we used transmission electron microscopy (TEM) and cryo-TEM. These techniques have been successfully used to study the morphology and the mechanism of assembly of amyloids.^31–34^ Hybrid β-hairpin **1** was studied both in water and in methanol.

**Fig. 6:**
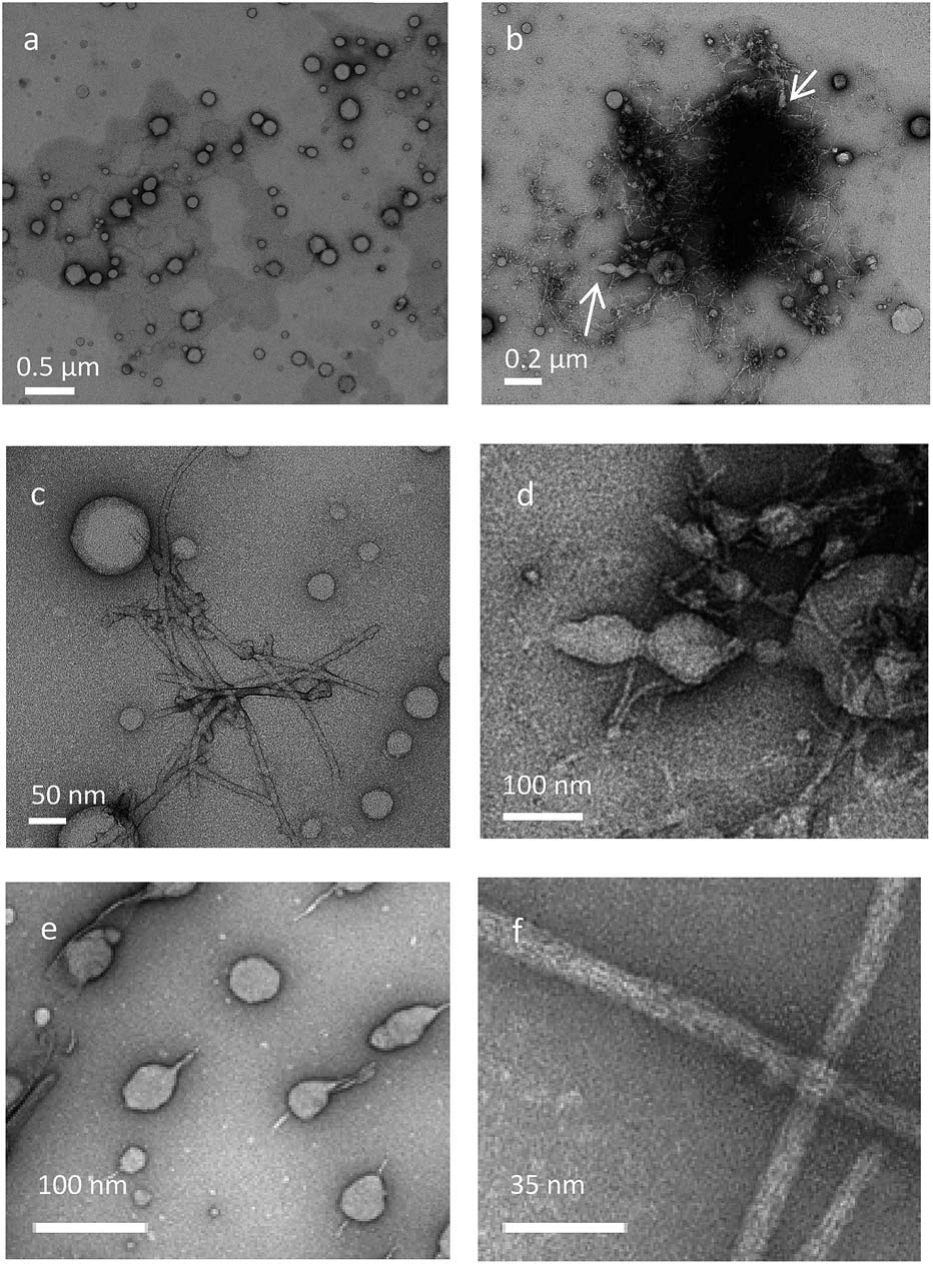
TEM images for the different stereoisomers of **1**. (a) TEM image for *trans*-**1** in methanol. (b and c) TEM image for stable *cis*-**1**. Arrows indicate the transition state from vesicles to fibers. (d) Zoom of TEM image b. (e and f) Native amyloid β, Aβ(16–22) in buffer. (e and f) were adapted with permission from ref. 31. Copyright 2012 American Chemical Society.

In methanol, *trans*-**1** forms vesicles (Fig. 6a), while the stable *cis*-**1** shows the formation of different aggregates, vesicles and fibers (Fig. 6b and c). The fibers are similar to those formed by amyloidogenic peptides (Fig. 6f).^17,33–35^ The presence of different aggregates, vesicles and fibers, can explain the coexistence of patterns of signals for the same amino acid in the NMR studies. Probably the hybrid peptide goes through a phase transition phenomenon: the fibers are formed from vesicles as recently reported, for example, for the peptide Ac-KLVFFAE-NH_2_.^31^ In our system it is possible to visualize this transition state, indicated with an arrow in Fig. 6b and detailed in Fig. 6d, consistent with native amyloid Aβ(16–22) (Fig. 6e).^31^

**Fig. 7:**
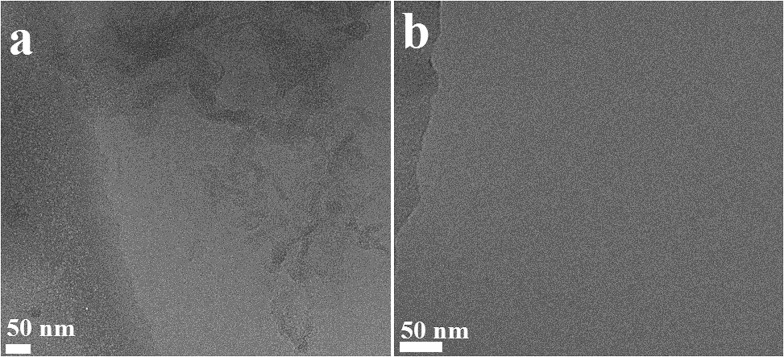
Cryo-TEM images for the different stereoisomers of **1**. (a) Cryo-TEM image for *trans*-**1** (1 mg mL^–1^) in water. (b) Cryo-TEM image for stable *cis*-**1** (1 mg mL^–1^) in water.

In water, *trans*-**1** shows strong aggregation behaviour, as shown by cryo-TEM (Fig. 7a) and sheet-like structures are formed. The irradiation-promoted isomerisation to unstable *cis*-**1** (Fig. S15, ESI†) and subsequent heating, to form stable *cis*-**1**, disrupt this sheet-like structure, and almost no aggregation is observed by cryo-TEM (Fig. 7b). The aggregation of *trans*-**1** can be explained by comparison to a system where β-hairpin was modified with *trans*-azobenzene.^10,12^ The system in the *trans* form is flexible enough to be able to adopt an extended conformation. This facilitates interfibrillar cross-linking.^10^ Although it is clear that in both cases the hybrids form interconnected structures, the aggregates look different in cryo-TEM images compared to the ones found by other groups:^10,16^ the overcrowded alkene photoswitch induces sheet-like structures (Fig. 7a) and not fibril-like structure as seen with azobenzene peptide.^10,16^ The sheet-like structures are probably formed due to the fact that the two strands of different molecules interact, but we believe that also the highly hydrophobic core of the overcrowded alkene promotes aggregation in a different manner than azobenzene (Fig. 7). We postulate that the structural differences in the two solvents, methanol and water, manifest due to the high hydrophobicity of the overcrowded alkene switch: probably in water the tendency of aggregate is much stronger.

**Fig. 8:**
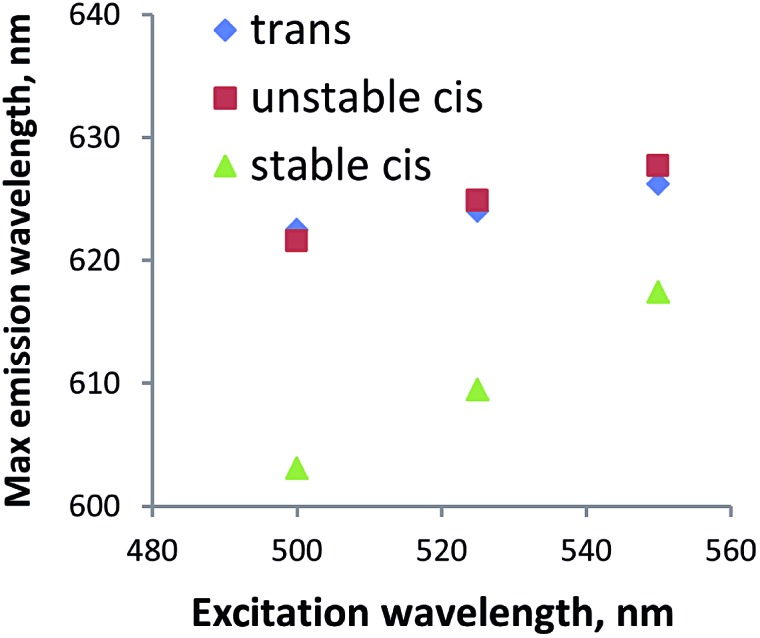
Nile red experiment. Plot of the excitation wavelengths (500 nm, 525 nm, 550 nm) *versus* the emission wavelengths maxima for *trans*-**1** (1 mg mL^–1^ in water), unstable *cis*-**1** (1 mg mL^–1^ in water) and stable *cis*-**1** (1 mg mL^–1^ in water) after addition of 2 μL Nile red solution (0.25 mM in ethanol).

To further study if peptide **1** in the *cis* configuration forms aggregates in water, fluorescence measurements were performed using Nile red. This dye, which is often used for studying lipids,^36^ is a very sensitive probe for domains that differ in polarity.^37^ If the system is homogeneous, the probe is constantly in the same environment; with one species present, the change in excitation wavelength does not cause a change in emission wavelength. If the probe is exposed to environments of distinct polarity, *e.g.* due to the existence of hydrophobic aggregates in the solution, the protonation state of the probe is different which leads to the co-existence of species with different fluorescence properties. Therefore, by changing the wavelength of excitation, multiple species will be addressed, which will lead to the change in emission spectra. For compound **1** in all the stereoisomers, the maximum wavelength of emission changes with the excitation wavelength (Fig. 8). Therefore we conclude that two *cis* forms, as well as the *trans* form, are indeed forming apolar aggregates in water.

## Conclusions

An overcrowded alkene molecular switch that can be used as a building block for solid phase peptide synthesis was designed, synthesized and used as a responsive unit in a β-hairpin-forming peptide. It demonstrated to be an excellent photoresponsive element to control biomolecule structure and organisation: even when inserted in the peptide, the light- and heat-induced transformations from *trans*-**1** to unstable *cis*-**1** and to stable *cis*-**1** are very selective processes characterized by high conversions. Notably, the unstable *cis* stereoisomer offers a different chiral environment than the stable *cis* form and, due to its metastability, it could be used as a tool for the study of dynamics of secondary structure formation. The photochemistry, isomerisation cycle, secondary structure and aggregation of **1** were studied in detail in methanol. CD and NMR showed that *trans*-**1** does not form any ordered structures, while the unstable and stable *cis*-**1** show the characteristic behaviour of peptides that adopt a β-hairpin structure. At higher concentration, the *trans* stereoisomer forms vesicles but, in the *cis* forms, co-existing structures are observed in NMR. The TEM measurements confirmed that different aggregates, fibers and vesicles, are present simultaneously. Transition phase aggregates could also be observed. Importantly, *cis*-**1** in methanol forms structures typically associated with amyloidogenic peptides.^31^

In water, hybrid peptide **1** also forms aggregates, as observed by cryo-TEM. In particular, *trans*-**1** shows sheet-like structures that can be disrupted by irradiation. The *cis* stereoisomers seem to form aggregates invisible by TEM, but verified by Nile red experiment, although simple precipitation cannot be excluded. In the future this system could also be studied using LD,^38^ which might provide additional information on the aggregates.

Interestingly, the aggregates formed by the *trans* stereoisomer are very different from the ones obtained by modifying the same β-hairpin with an azobenzene.^10^ In general, the spatial arrangement that the three stereoisomers of the overcrowded alkene switch provoke might be different than the one given by azobenzene switches.^10,13,16^ The introduction of this overcrowded alkene switch, therefore, opens new possibilities not only to study biological processes, but also to create new bio-compatible and bio-inspired tunable materials, taking advantage of the bistability of the overcrowded alkene switch.
