# Supplementary material for: Light and heat control over secondary structure and amyloid-like fiber formation in an overcrowded-alkene-modified Trp zipper
Source: Chem Sci. 2015 Sep 23;6(12):7311–8. doi: 10.1039/c5sc02735g (PMC5512536; doi:10.1039/c5sc02735g)
Supplement: Supplementary file 1 [file SC-006-C5SC02735G-s001.pdf]

Electronic Supplementary Material (ESI) for Chemical Science.
This journal is © The Royal Society of Chemistry 2015

Supporting Information

Light and heat control over secondary structure and amyloid fiber formation of an overcrowded-alkene-modified Trp zipper

Claudia Poloni[a], Marc Stuart[a], Pieter van der Meulen[a], Wiktor Szymanski[b]*, Ben L. Feringa[a]*

[a]Centre for Systems Chemistry, Stratingh Institute for Chemistry, Faculty of Mathematics and Natural Sciences, University of Groningen, Nijenborgh 4, 9747AG Groningen, The Netherlands. b.l.feringa@rug.nl; Fax: (+31) 50-3634279.
[b]Department of Radiology, University of Groningen, University Medical Center Groningen, Hanzeplein 1, 9713 GZ, Groningen, The Netherlands. w.c.szymanski@rug.nl.

Synthesis of overcrowded alkene switch, building block for solid phase peptide synthesis 2

NMR Spectra 8

Isomerisation cycle of the compound 5 12

Isomerisation cycle of compound 1 16

CD Spectroscopy for compound 3 18

CD Spectroscopy for compound 1 19

CD Spectroscopy for compound 8 22

2D-NMR Studies on compound 1 23

TEM pictures for compound 1 31

Cryo-TEM pictures for compound 1 31

Nile red experiment 36

Synthesis of overcrowded alkene switch, building block for solid phase peptide synthesis

All chemicals for the synthesis were obtained from commercial sources and used as received unless stated otherwise. Solvents were reagent grade. Thin-layer chromatography (TLC) was performed using commercial Kieselgel 60, F254 silica gel plates, and components were visualized with $KMnO_4$ or phosphomolybdic acid reagent. Flash chromatography was performed on silica gel (Silicycle Siliaflash P60, 40-63 m, 230-400 mesh). Drying of solutions was performed with $MgSO_4$ and solvents were removed with a rotary evaporator. Chemical shifts for NMR measurements were determined relative to the residual solvent peaks ($CHCl_3$, δ = 7.26 ppm for hydrogen atoms, δ = 77.0 for carbon atoms). The following abbreviations are used to indicate signal multiplicity: s, singlet; d, doublet; t, triplet; q, quartet; m, multiplet; br s, broad signal. 2D NMR spectra were recorded in CD_3OH at 20 °C and 5 °C on a Agilent 600. The 2D TOCSY spectra were recorded with a spin-lock period of 70 ms. 2D NOESY spectra were recorded with mixing times of 400 to 600 ms. HRMS (ESI) spectra were obtained on a Thermo scientific LTQ Orbitrap XL. MALDI spectra were obtained on a MALDI/TOFTOF 4800 by AB Sciex; the analysis was done in positive mode using the matrix alpha-cyano-hydroxycinnamic acid. Solid phase peptide synthesizer CEM Liberty, with CEM Discover microwaves was used for solid phase peptide synthesis. Optical rotations were measured on a Schmidt + Haensch polarimeter (Polartronic MH8) with a 10 cm cell (c given in g/100 mL) at 20 °C. Melting points were recorded using a Buchi melting point B-545 apparatus. UV/Vis absorption spectra were recorded on an Agilent 8453 UV-Visible Spectrophotometer using Uvasol-grade solvents. CD spectra were recorded on a CD spectrophotometer JASCO J815. Irradiation experiments were performed with a spectroline ENB-280C/FE UV lamp (312 nm). RP-HPLC was carried out with Shimadzu equipment using a linear gradient of 1.54% of eluent A per min at a flow rate of $0.5 mLmin^{-1}$. The eluents A and B are 0.1 % TFA acetonitrile and 0.1 % $_{aq}$TFA, respectively. For analytical RP-HPLC, a XTerra C18 3.0x150mm column (Waters) was used and for semi-preparative RP-HPLC, a XTerra Prep C18 7.8x150mm column (Waters) was used.

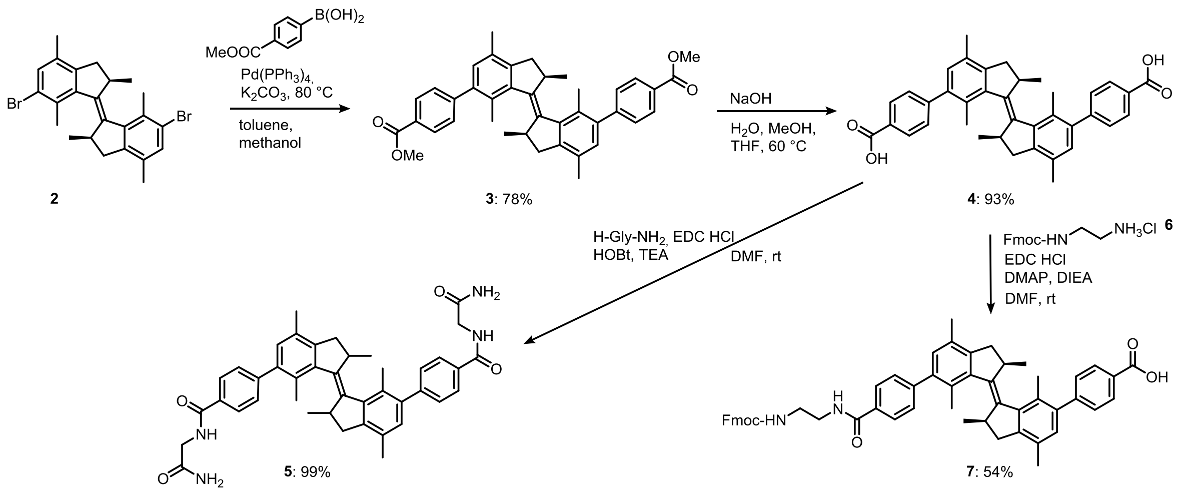

Scheme S1: Synthesis of compounds **5** and **7**.

The enantioselective synthesis of **2** was achieved following a published procedure.[1] The experimental data are in accordance with Ref. 1.

Compound **6** was synthesized according to literature procedure.[2] The experimental data are in accordance with Ref. 2.

Synthesis of 3

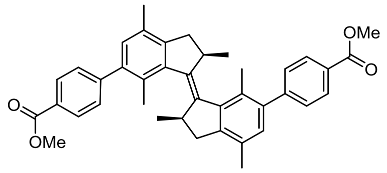

Compound **2** (243 mg, 0.51 mmol, 1 eq), potassium carbonate (493 mg, 7 eq), tetrakis(triphenylphosphine)palladium (47 mg, 8 mol%) and 4-(methoxycarbonyl)phenylboronic acid (275 mg, 3 eq) were dissolved in dry toluene:methanol 3:1 in a three necked flask equipped with a reflux condenser. The reaction mixture was stirred for 24 h at 90°C under N_2 atmosphere. After cooling down to rt, water was added. The aqueous layer was washed twice with CH_2Cl_2. The collected organic solution was dried with $MgSO_4$ and the solvent was removed under vacuum. The crude product was purified by flash-chromatography (silica, pentane: CH_2Cl_2, 1:1). Product **3** (*trans*:*cis* mixture) was obtained as a white powder in 74% yield. The two stereoisomers were separated (*trans*:*cis* ratio 4:1) by column chromatography (silica, pentane: CH_2Cl_2, 1:1). *Trans*-**3** was isolated (151 mg, 0.25 mmol). m.p.: 204-206 °C; $[\alpha]_D^{20}$=+170° ($CHCl_3$, c=0.1); ^{1}H NMR (400 MHz, $CDCl_3$): δ 8.11 (m, 4H, arom), 7.50 (m, 4H, arom), 6.96 (s, 2H, arom), 3.96 (s, 6H, 2 C**H**$_3$OCO), 3.08 – 2.98 (m, 2H, 2 C**H**CH$_3$), 2.78 (dd, *J* = 14.5, 5.5 Hz, 2H, C**H**$_2$CHCH$_3$), 2.36 – 2.24 (m, 8H, C**H**$_2$CHCH$_3$, 2 C**H**$_3$ ar), 1.13 (d, *J* = 6.5 Hz, 6H, CHC**H**$_3$). ^{13}C NMR (400 MHz, $CDCl_3$): δ 167.4, 147.8, 142.6, 142.3, 140.3, 131.6, 129.8, 129.7, 129.6, 128.7, 128.6, 53.6, 52.2, 42.7, 39.2, 21.4, 19.3, 18.4. Expected ESI Mass ($C_{40}H_{41}O_4$): 585.2999 [M+H$^+$]. Found: 585.2995.

[1] Neubauer M. T., van Leeuwen T., D. Zhao D., Lubbe A. S., Kistemaker J. C. M., and Feringa B. L., *Org. Lett.*, **2014**, 16, 4220–4223.
[2] Boeijen A., van Ameijde J., Liskamp R. M. J., *J. Org. Chem.*, **2001**, 66, 8454-8462.

Synthesis of *trans*-4

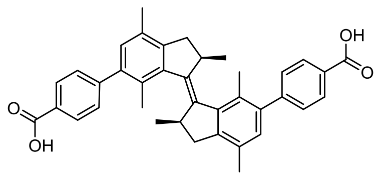

Trans-**3** (50 mg, 0.08 mmol, 1 eq) was dissolved in a mixture of 5 mL THF and 3 mL methanol. 2M $_{aq.}$NaOH (300 μL) was added and the reaction mixture was stirred for 3 h at 60°C. After cooling down to rt, 1M $_{aq.}$HCl was added drop-wise till pH=2. The resulting mixture was extracted twice with CH_2Cl_2. The collected organic layer was dried with $MgSO_4$ and the solvent removed under vacuum. *Trans*-**4** was isolated as a white powder (42 mg, 0.075 mmol, yield: 93%). m.p.: 282-284 °C (dec); $[\alpha]_D^{20}$=+124° (CH_3OH, c=0.1); ^{1}H NMR (400 MHz, DMSO-d_6) δ 8.02 (m, 4H, arom), 7.57 (m, 4H, arom), 6.98 (s, 2H, arom), 2.99 (m, 2H, C**H**CH_3), 2.69 (dd, *J* = 14.5, 5.8 Hz, 2H, C**H**$_2$CHCH$_3$), 2.34 (d, *J* = 14.5 Hz, 2H, C**H**$_2$CHCH$_3$), 2.26 (s, 6H, C**H$_3$** ar), 2.22 (s,6H, C**H$_3$** ar), 1.07 (d, *J* = 6.0 Hz, 6H, CHC**H$_3$**). ^{13}C NMR (400 MHz, DMSO) δ 167.2, 146.3, 141.8, 141.6, 139.7, 131.3, 129.6, 129.5, 129.2, 129.1, 127.8, 41.8, 38.5, 29.0, 21.0, 19.0, 17.9. Expected ESI Mass ($C_{38}H_{37}O_4$): 557.2686 [M+H$^+$]. Found: 557.2686.

Synthesis of 7

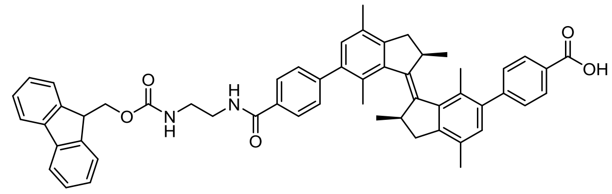

Trans-**4** (35 mg, 0.063 mmol, 1.1 eq) was dissolved in 1 mL DMF:CH_2Cl_2 (1:1) and compound **6** (18 mg, 0.057 mmol, 1 eq), EDC·HCl (12 mg, 0.063 mmol, 1.1 eq), DMAP (0.5 mg, 5 mol%) and DIPEA (17 uL, 0.1 mmol, 1 eq) were added. After 48 h, 20 mL DCM was added and the organic phase was washed with 1N $_{aq.}$HCl solution (2 x 20 mL) and brine (2 x 20 mL). The organic layer was dried and the solvent evaporated. The product was purified by column chromatography (silica, DCM:MeOH 4:0.1). *Trans*-**7** was isolated as a white powder (25 mg, 0.030 mmol, yield= 54%). m.p. 196-198 °C; ^{1}H NMR (400 MHz, $CDCl_3$) δ 8.19 (m, 2H, arom), 7.87 (m, 2H, arom), 7.75 (d, *J* = 7.5 Hz, 2H, arom Fmoc), 7.58 (d, *J* = 7.5 Hz, 2H, arom Fmoc), 7.54 (m, 2H, arom), 7.47 (m, 2H, arom), 7.38 (t, *J* = 7.3 Hz, 2H, arom Fmoc), 7.28 (t, *J* = 7.4 Hz, 2H, arom Fmoc), 7.05 (s, 1H, CH_2N**H**CO), 6.97 (s, 1H, arom), 6.92 (s, 1H, arom), 5.40 (m, 1H, CH_2N**H**COO), 4.43 (d, *J* = 7.0 Hz, 2H, Fmoc CHC**H$_2$**), 4.21 (t, *J*=7.0 Hz, 1H, Fmoc C**H**CH_2), 3.66 (m, 2H, C**H**$_2$NH), 3.52 (m, 2H, C**H**$_2$NH), 3.02 (m, 2H, C**H**CH_3), 2.77 (dd, *J* = 14.4 Hz, 5.4 Hz, 2H, C**H**$_2$CHCH$_3$), 2.30 (m, 14H, C**H**$_2$CHCH$_3$, C**H$_3$** ar), 1.12 (m, 6H, CHC**H$_3$**). ^{13}C NMR (400 MHz, $CDCl_3$) δ 170.5, 168.2, 157.9, 148.5, 146.3, 143.9, 143.9, 142.6, 142.4, 142.3, 142.3, 142.3, 142.1, 141.4, 140.3, 140.2, 132.3, 131.6, 131.5, 130.2, 129.9, 129.7, 129.6, 128.9, 128.6, 127.9, 127.2, 127.0, 125.2, 120.1, 67.2, 47.4, 42.6, 41.4, 41.0, 39.2, 32.0, 29.8, 29.5, 22.8, 21.3, 19.2, 19.2, 18.4, 18.4, 14.3. Expected ESI Mass ($C_{55}H_{53}N_2O_5$): 821.3949. Found: 821.3955.

Synthesis of *trans*-5

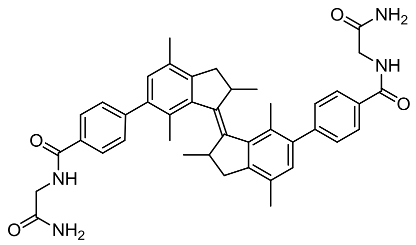

To a solution of racemic *trans*-**4** (73 mg, 0.13 mmol, 1 eq) in 1 mL DMF: CH_2Cl_2 (1:1), EDC·HCl (49 mg, 0.26 mmol, 2 eq) and HOBt (4.7 mg, 2 eq) were added. After 30 min, glycinamide hydrochloride (8.5 mg, 2 eq and TEA (35 uL, 2 eq) were added. After 24 h, 50 mL AcOEt was added and the organic phase was washed with 1N $_{aq.}$HCl solution (2 x 50 mL), brine (1 x 50 mL), $_{sat.aq.}$$NaHCO_3$ solution (2 x 50 mL) and again brine (1 x 50 mL). The organic layer was dried and the solvent was evaporated. The product was isolated as a white powder (45 mg , 0.067 mmol, yield = 56%). m.p. 210 °C (dec.); ^{1}H NMR (400 MHz, DMSO-d_6) δ, 8.22 (2H, 2 N**H**), 7.97 (m, 4H, arom), 7.55 (m, 4H, arom), 7.39 (s, 2H, N**H**$_2$), 7.04 (s, 2H, N**H**$_2$), 6.97 (s, 2H, arom), 3.84 (d, 4H, C**H**$_2$ Gly), 3.12 – 2.97 (m, 2H, C**H**CH_3), 2.55 (m, 2H, C**H**$_2$CHCH_3), 2.26 (m, 2H, C**H**$_2$CHCH_3), 2.26 (s, 6H, C**H$_3$** ar), 2.23 (s, 6H, C**H$_3$** ar), 1.08 (d, *J* = 6.4 Hz, 6H, CHC**H$_3$**). ^{13}C NMR (400 MHz, DMSO-d_6) δ 171.1, 166.2, 144.8, 141.6, 141.6, 139.9, 132.4, 131.2, 129.5, 129.2, 127.8, 127.3, 42.5, 41.8, 38.5, 29.0, 21.0, 19.1, 17.9. Expected ESI Mass ($C_{42}H_{45}N_4$ O_4): 669.3435. Found: 669.3434.

Synthesis of 1: Ac-Ser-Trp-Thr-Trp-Glu-switch-Lys-Trp-Thr-Trp-Lys-NH_2

The switch-peptide hybrid **1** was synthesized on a 0.009 mmol scale by standard protocol of Fmoc chemistry SPPS (Solid Phase Peptide Synthesis). Sieber resin was used (0,69 mmol/g). Fmoc-Trp-OH, Fmoc-Lys(Trt)-OH, Fmoc-Thr(Trt)-OH, Fmoc-Glu(O-2-PhiPr)-OH, Fmoc-Ser(Trt)-OH were used. The coupling steps were performed with 5 eq Fmoc-protected amino acid, 5 eq HBTU and 10 eq DIPEA (2 x 45 min). The Fmoc-deprotection step was performed with 20% piperidine in DMF (1 x 30 min). The acetylation step was performed with 10 eq Ac_2O, 0.1 eq HOBt and 10 eq DIPEA. Cleavage from the resin was performed at 0 °C for 1 h with TFA:DCM (1:3) with 2.5 $_{vol}$% 2,2-(ethylenedioxy)diethanethiol, 2.5 $_{vol}$% H_2O, 1 $_{vol}$% TIS. The crude peptide was purified by RP-HPLC on C18 semi-preparative column. Purity: 96%. Ret. Time: 32.56 min.

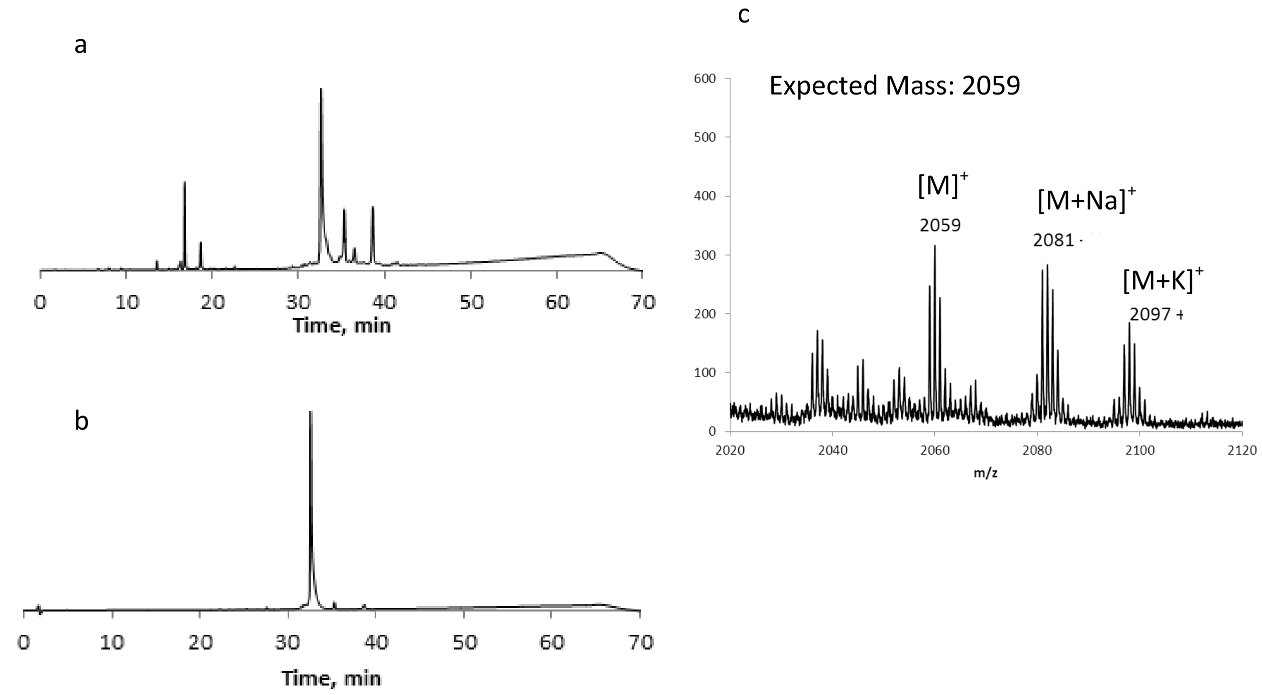

Figure S1. HPLC traces (detection wavelength: 220 nm) for a) crude product **1** and b) purified product **1**. c) MALDI-TOF spectrum for the product.

8: Ac-Ser-Trp-Thr-Trp-Glu-Gly-Asn-Lys-Trp-Thr-Trp-Lys-NH_2

The peptide was synthesized on a 0.1 mmol scale by standard protocol of Fmoc chemistry SPPS (Solid Phase Peptide Synthesis). Sieber resin was used (0,65 mmol/g). Fmoc-Trp-OH, Fmoc-Lys(Trt)-OH, Fmoc-Thr(Trt)-OH, Fmoc-Glu(OtBu)-OH, Fmoc-Ser(Trt)-OH, Fmoc-Gly-OH, Fmoc- Asn(Trt)-OH were used. The coupling step was performed with 5 eq Fmoc-protected amino acid, 5 eq HBTU and 10 eq DIPEA (2 x 45 min). The Fmoc-deprotection step was performed with 20% piperidine in DMF (1 x 30 min). Product **8** was obtain from the peptide synthesizer. The last Fmoc-deprotection step was performed with 20% piperidine in DMF (1 x 30 min). The acetylation step was performed with 10 eq Ac_2O, 0.1 eq HOBt and 10 eq DIPEA. Cleavage from the resin was performed at 0 °C for 1 h with 95% TFA, 2.5 $_{vol}$% tri-*iso*-propylsilane (TIS), 2.5 $_{vol}$% H_2O. The crude peptide was purified by RP-HPLC on C18 semi-preparative column. Purity: 98%. Ret. Time: 21.02 min.

a

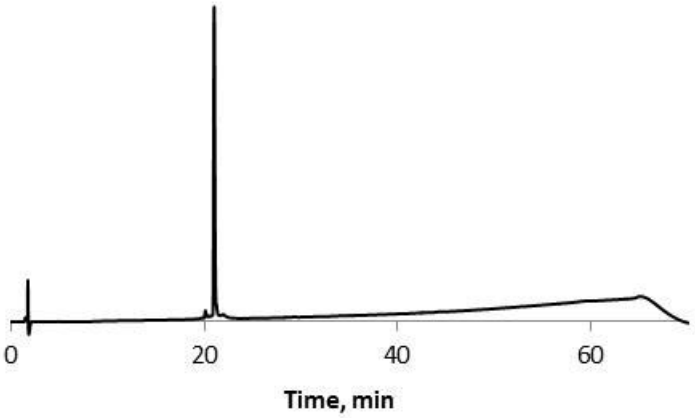

b

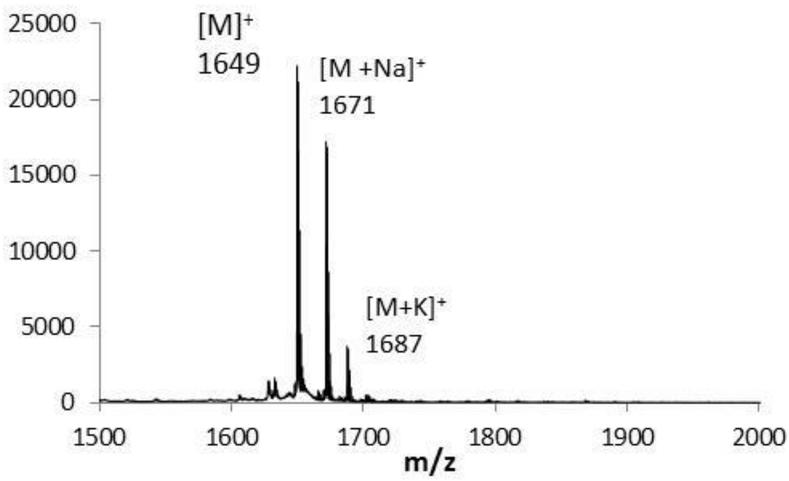

Figure S2. a) HPLC trace (detection wavelength: 220 nm) for purified product **8**. b) MALDI-TOF spectrum for the product.

NMR Spectra

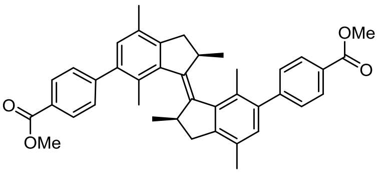
OMe
O
O
OMe

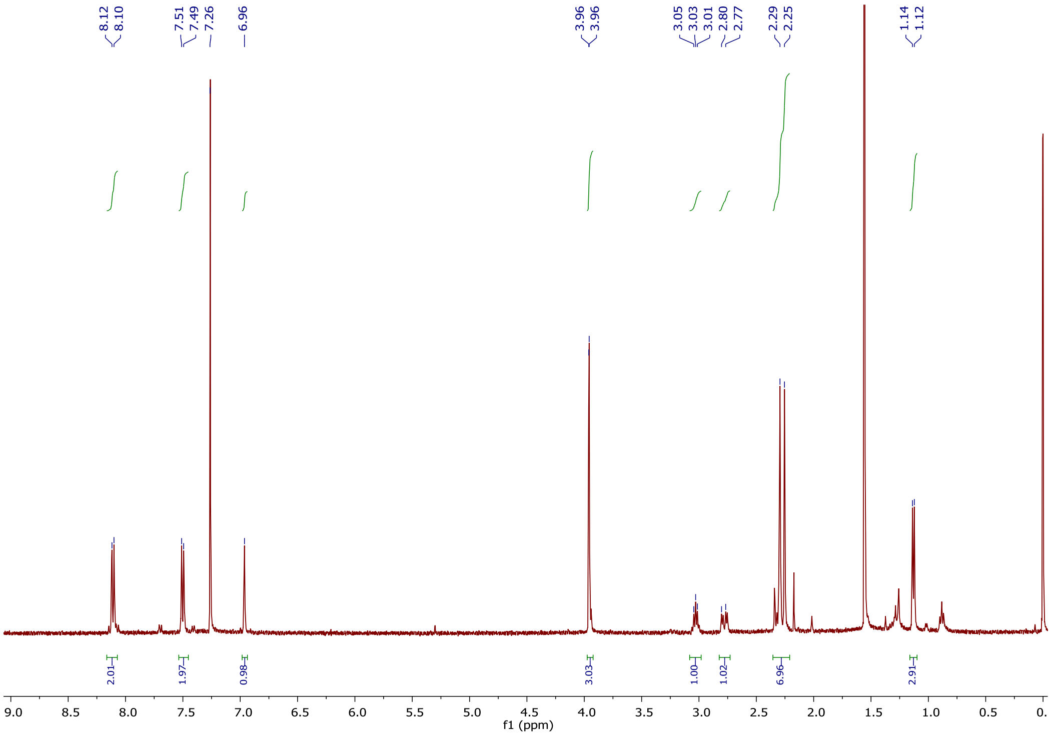
8.12
8.10
7.51
7.49
7.26
6.96
3.96
3.96
3.05
3.03
3.01
2.80
2.77
2.29
2.25
1.14
1.12
2.01
1.97
0.98
3.03
1.00
1.02
6.96
2.91
f1 (ppm)

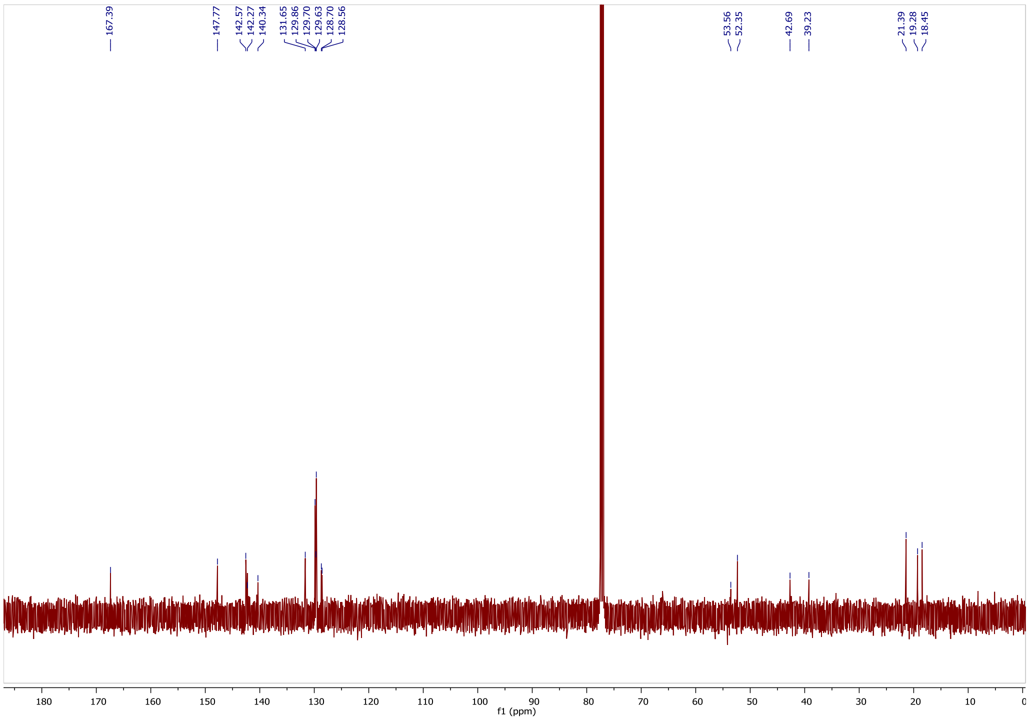
167.39
147.77
142.57
142.27
140.34
131.65
129.86
129.70
129.63
128.70
128.56
53.56
52.35
42.69
39.23
21.39
19.28
18.45
f1 (ppm)

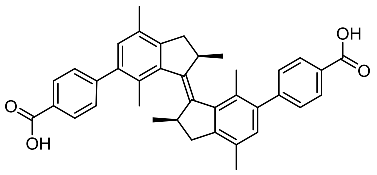
OH
O
O
OH

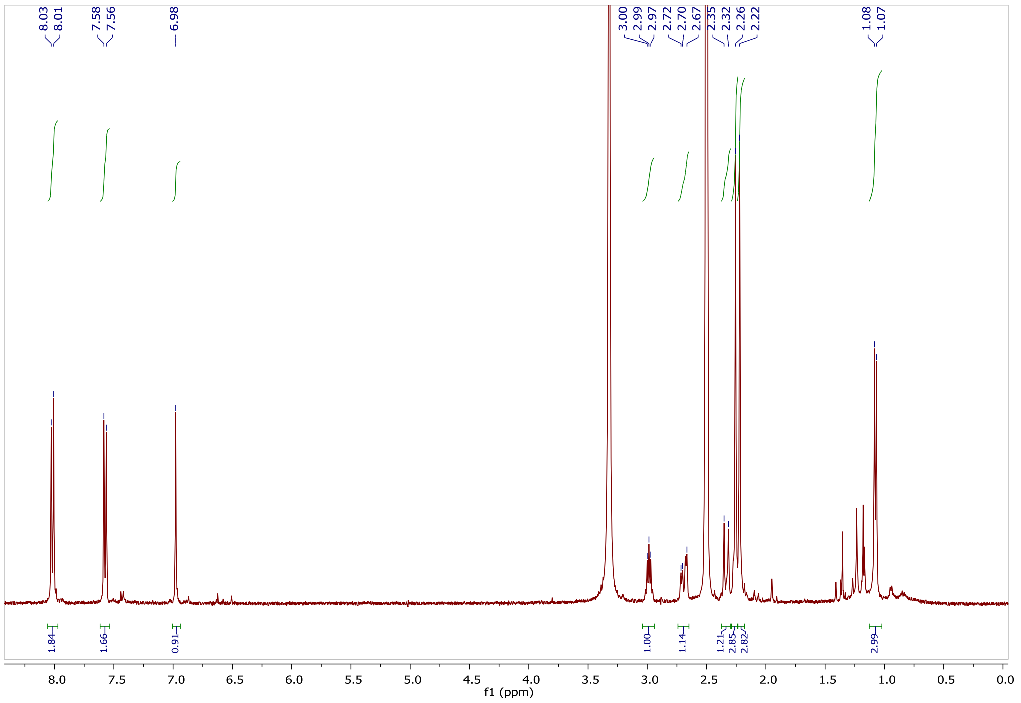
8.03
8.01
7.58
7.56
6.98
3.00
2.99
2.97
2.72
2.70
2.67
2.35
2.32
2.26
2.22
1.08
1.07
1.84
1.66
0.91
1.00
1.14
1.21
2.85
2.82
2.99
f1 (ppm)

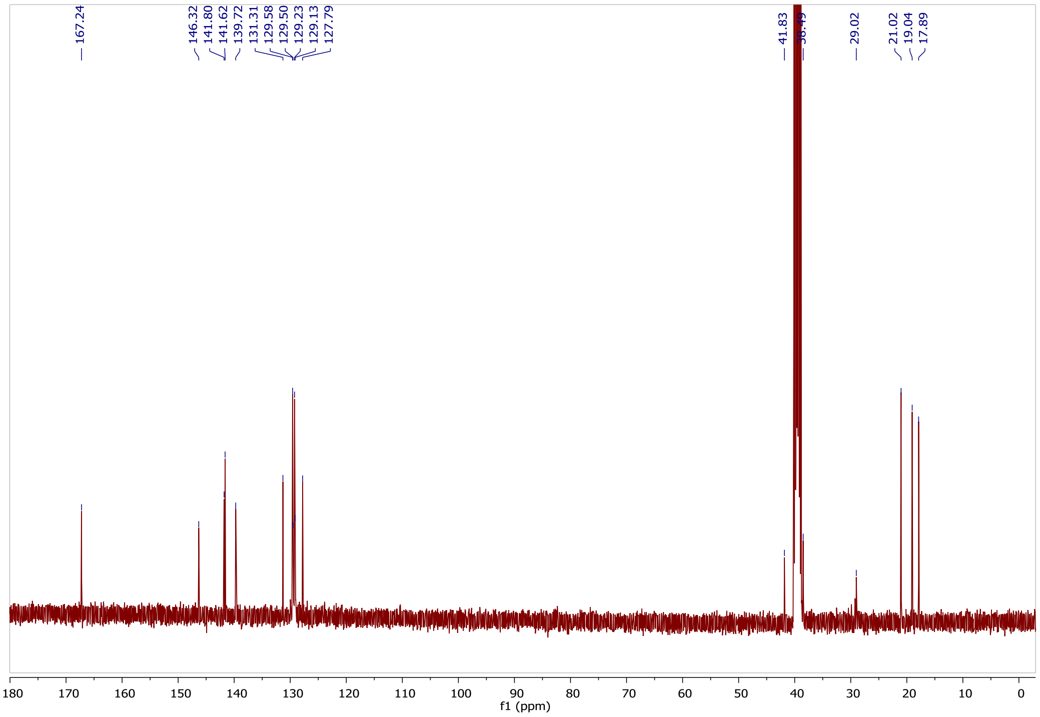
167.24
146.32
141.80
141.62
139.72
131.31
129.58
129.50
129.23
129.13
127.79
41.83
29.02
21.02
19.04
17.89
f1 (ppm)

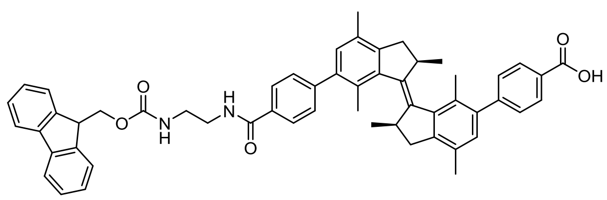
O
O
N
H
H
N
O
O
OH

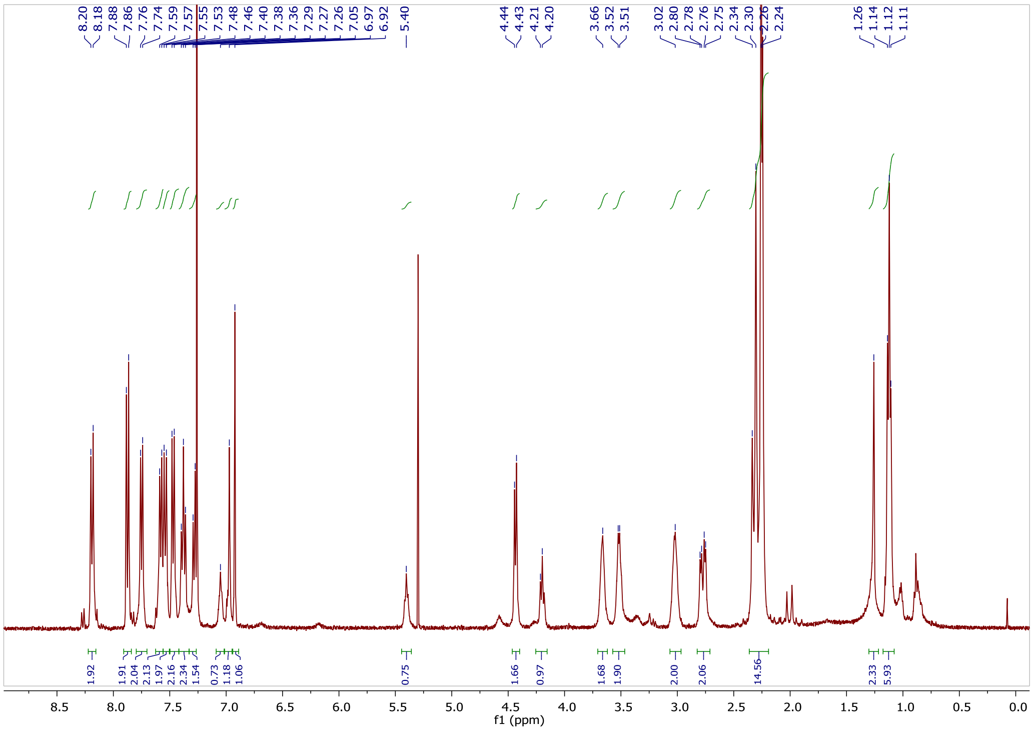
8.20
8.18
7.88
7.86
7.76
7.74
7.59
7.57
7.55
7.53
7.48
7.46
7.40
7.38
7.36
7.29
7.27
7.26
7.05
6.97
6.92
5.40
4.44
4.43
4.21
4.20
3.66
3.52
3.51
3.02
2.80
2.78
2.76
2.75
2.34
2.30
2.26
2.24
1.26
1.14
1.12
1.11
1.92
1.91
2.04
2.13
1.97
2.16
2.34
1.54
0.73
1.18
1.06
0.75
1.66
0.97
1.68
1.90
2.00
2.06
14.56
2.33
5.93
8.5
8.0
7.5
7.0
6.5
6.0
5.5
5.0
4.5
4.0
3.5
3.0
2.5
2.0
1.5
1.0
0.5
0.0
f1 (ppm)

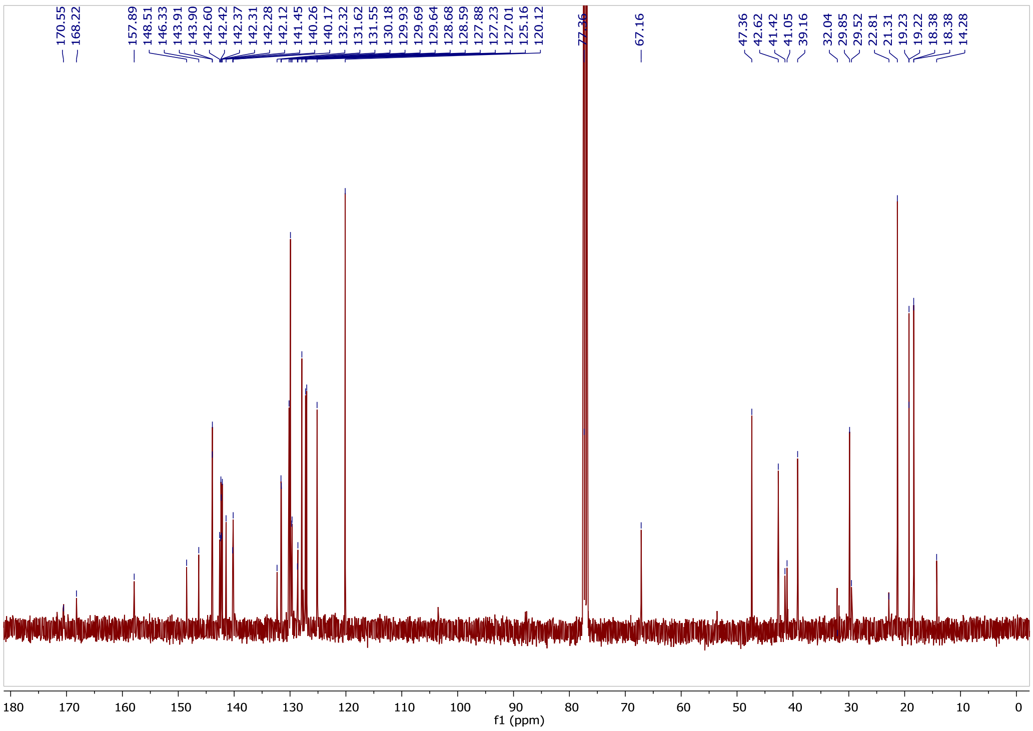
170.55
168.22
157.89
148.51
146.33
143.91
143.90
142.60
142.42
142.37
142.31
142.28
142.12
141.45
140.26
140.17
132.32
131.62
131.55
130.18
129.93
129.69
129.64
128.68
128.59
127.88
127.23
127.01
125.16
120.12
77.36
67.16
47.36
42.62
41.42
41.05
39.16
32.04
29.85
29.52
22.81
21.31
19.23
19.22
18.38
18.38
14.28
180
170
160
150
140
130
120
110
100
90
80
70
60
50
40
30
20
10
0
f1 (ppm)

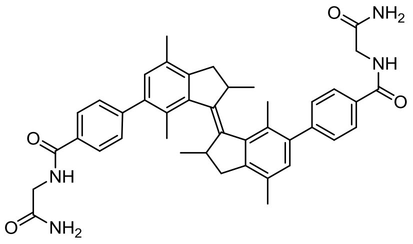
O
NH2
NH
O
O
NH
O
NH2

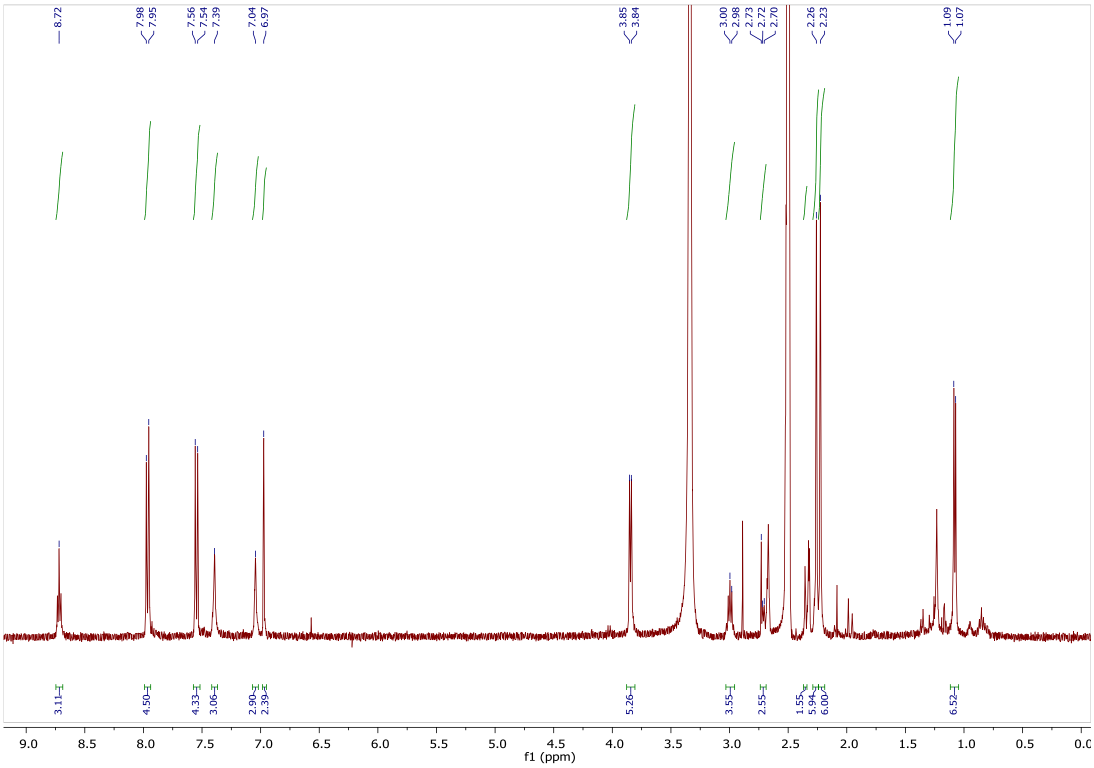
8.72
7.98
7.95
7.56
7.54
7.39
7.04
6.97
3.85
3.84
3.00
2.98
2.73
2.72
2.70
2.26
2.23
1.09
1.07
3.11
4.50
4.33
3.06
2.90
2.39
5.26
3.55
2.55
1.55
5.94
6.00
6.52
9.0 8.5 8.0 7.5 7.0 6.5 6.0 5.5 5.0 4.5 4.0 3.5 3.0 2.5 2.0 1.5 1.0 0.5 0.0
f1 (ppm)

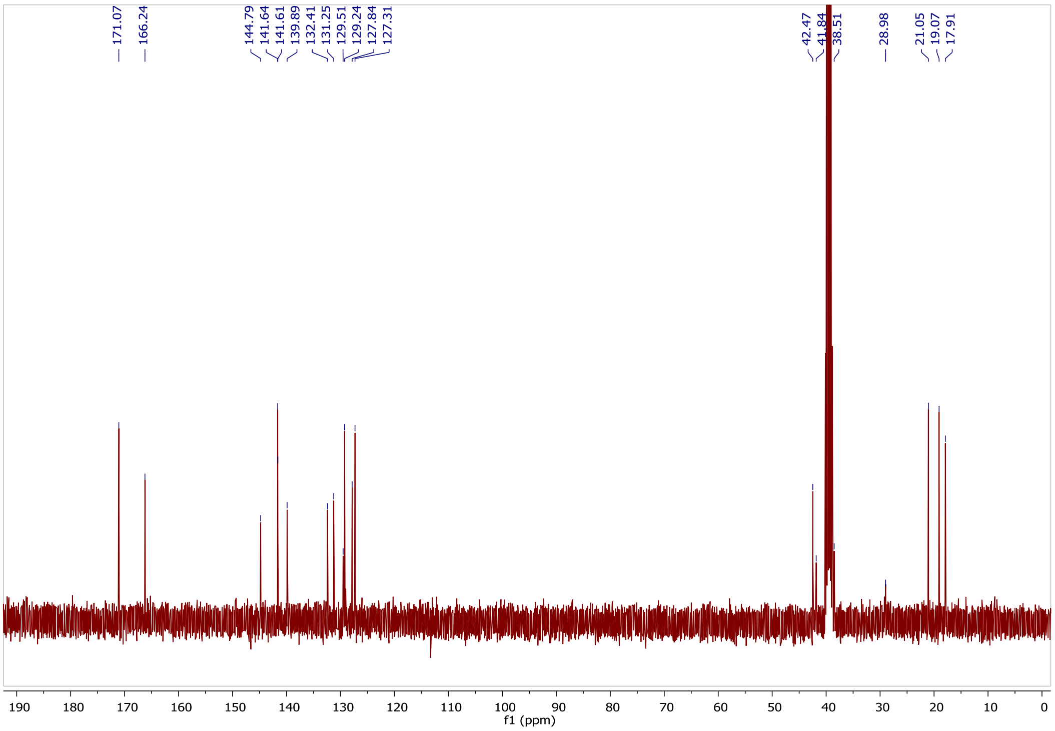
171.07
166.24
144.79
141.64
141.61
139.89
132.41
131.25
129.51
129.24
127.84
127.31
42.47
41.84
38.51
28.98
21.05
19.07
17.91
190 180 170 160 150 140 130 120 110 100 90 80 70 60 50 40 30 20 10 0
f1 (ppm)

Isomerization cycle of the compound 5

UV-vis and NMR spectroscopy were used to follow the isomerization steps of compound **5**. The cycle is shown in Fig. 3.

NMR was used to determine the PSS ratio (photostationary state) and the UV-vis spectroscopy to determine the $t_{1/2}$ and the thermodynamic parameters.

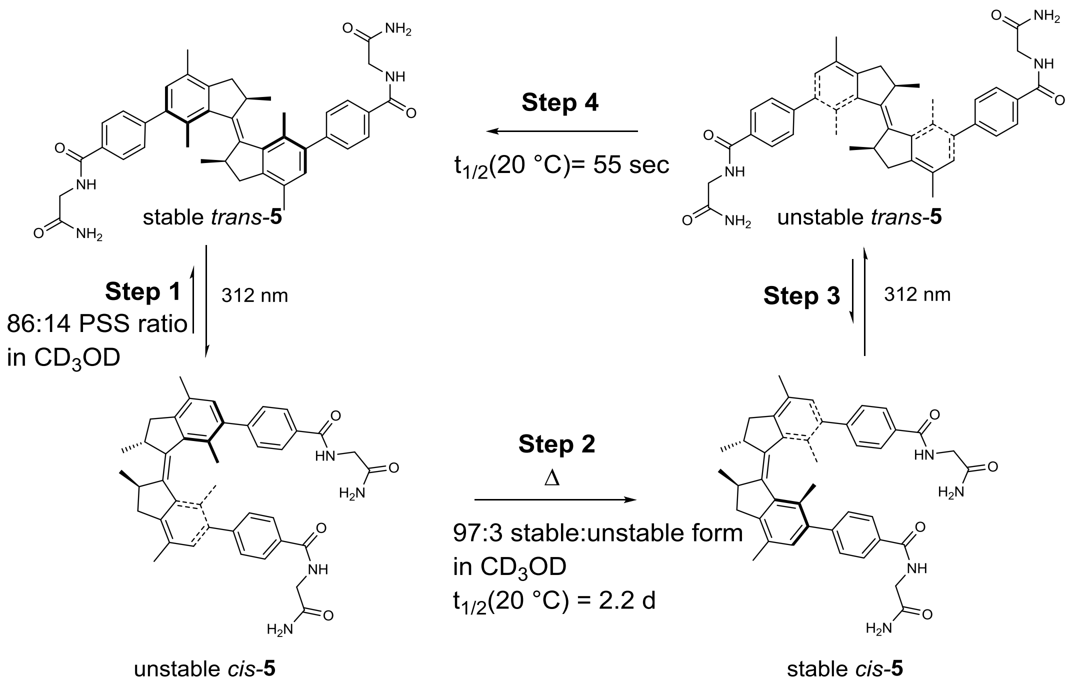

Figure S3. Isomerization cycle for compound **5**, half-life values and PSS ratio's.

NMR studies on compound 5

A solution of compound **5** (5 mg in 500 uL CD_3OD or DMSO-d_6) was irradiated with λ=312 nm until the photostationary state was reached. The PSS ratio stable *trans* - unstable *cis* isomerization is 14:86 in CD_3OD and 3:97 in DMSO. In CD_3OD, unstable *cis*-**5** precipitates from the solution. Afterwards the precipitate was filtered off and redissolved in CD_3OD to obtain a solution of unstable *cis*-**5** (3 mg in 300 uL) was prepared and warmed up at 40 °C.

Unstable *cis*-5: ^{1}H NMR (400 MHz, CD_3OD) δ 7.94 (d, *J* = 8.2 Hz, 4H, arom), 7.35 (d, *J* = 8.3 Hz, 4H, arom), 6.88 (s, 2H, arom), 4.05(s, 4H, C**H**$_2$ Gly), 3.62 (m, 2H, C**H**CH_3), 2.70 (dd, *J* = 15.8 Hz, 7.8 Hz, 2H, C**H**$_2$CHCH_3), 2.29 (s, 6H, C**H$_3$** ar), 1.53 (d, *J* = 6.0 Hz, 6H, CHC**H$_3$**), 1.36 (s, 6H, C**H$_3$** ar).

Stable *cis*-5: ^{1}H NMR (400 MHz, CD_3OD) δ 8.00 (d, *J* = 8.4 Hz, 4H, arom), 7.41 (d, *J* = 8.4 Hz, 4H, arom), 6.91 (s, 2H, arom), 4.08 (s, 4H, C**H**$_2$ Gly), 3.14 (dd, *J* = 14.9 Hz, 5.9 Hz, 2H, C**H**$_2$CHCH_3), 2.57 (d, *J* = 15.0 Hz, 2H, C**H**$_2$CHCH_3), 2.29 (s, 6H, C**H$_3$** ar), 1.45 (s, 6H, C**H$_3$** ar), 1.14 (d, *J* = 6.8 Hz, 6H, CHC**H$_3$**).

a

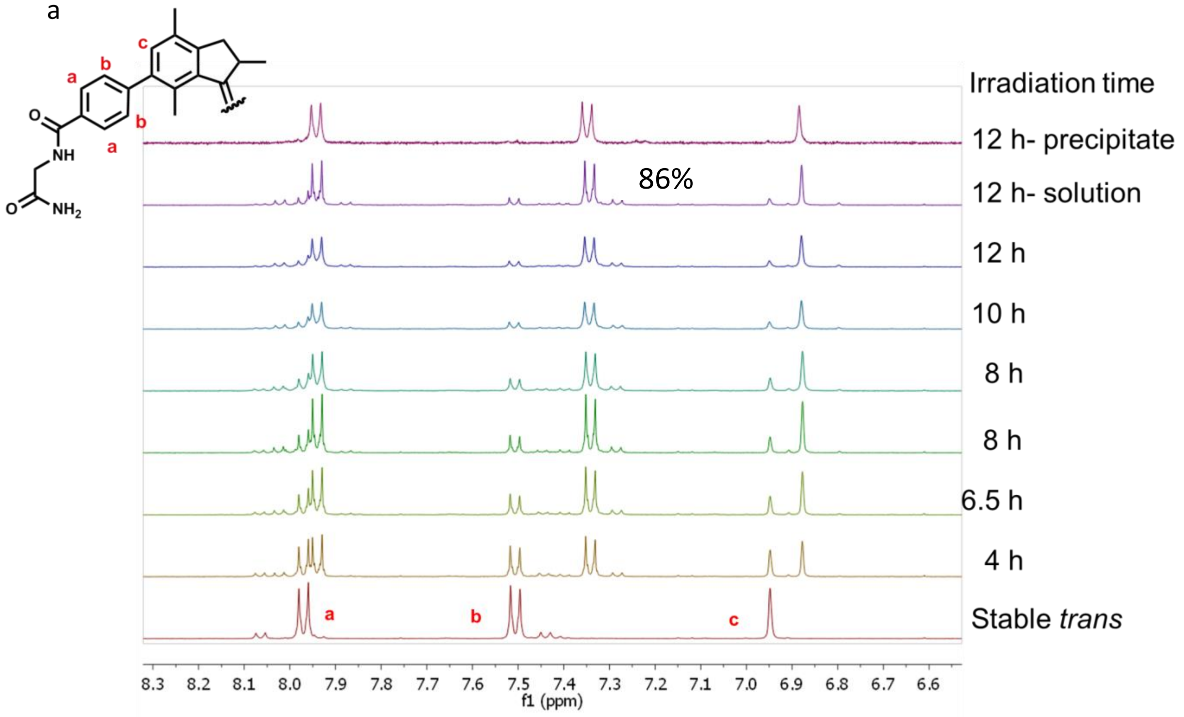

b

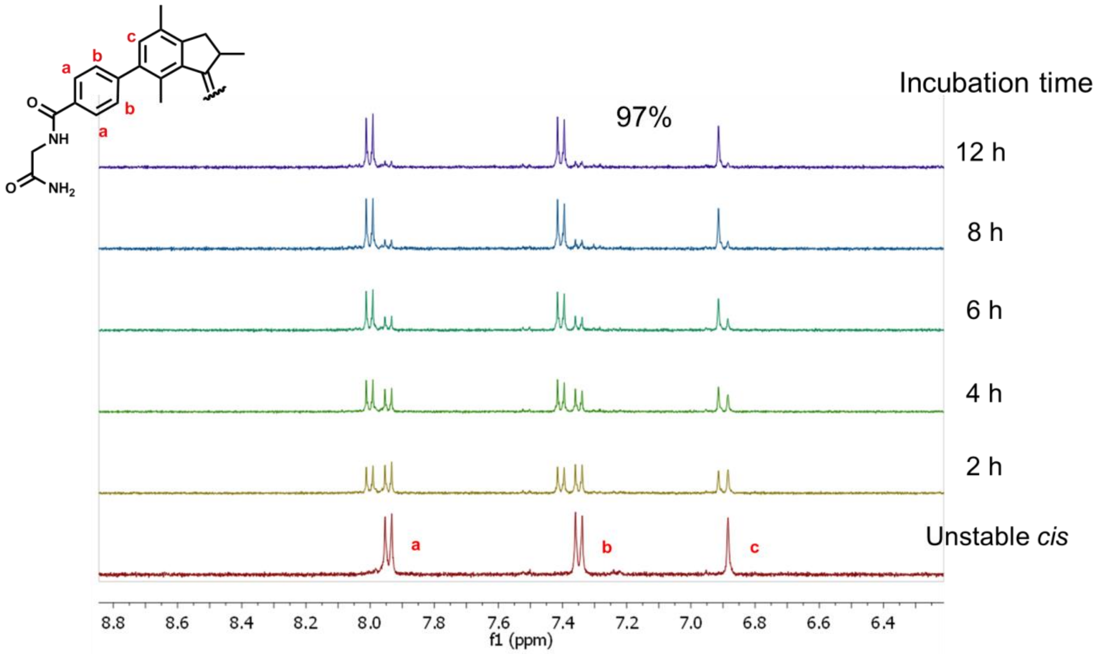

Figure S4. a) Irradiation at λ = 312 nm of *trans*-**5** (5 mg in 500 uL CD_3OD) followed in time by ^{1}H NMR (aromatic region). b) Thermal isomerization at 40 ˚C of unstable *cis*-**5** (3 mg in 300 uL) followed in time by ^{1}H NMR (aromatic region).

Kinetic study of compound 5 using UV-vis spectroscopy

A $1\cdot10^{-5}$ M solution of stable *trans*-**5** in MeOH was prepared and the spectrum of trans-**5** was recorded at 30 °C. The solution was irradiated at λ = 312 nm and the absorbance spectra were recorded till the photostationary state was reached (Fig. 5a). Next, a filter (cut-off λ = 340 nm) was positioned between the sample and the light source and the decrease in absorbance at λ = 350 nm was recorded. This permits, with a simple exponential decay curve, to calculate the rate constant, k. This procedure was repeated at 40 °C, 50 °C and 60 °C (Fig. 6a). Using the Eyring equation, the kinetic constants and the half-life time, $t_{1/2}$, of two thermal isomerization processes were calculated (Fig. 7a).

The same experiment was repeated for stable *cis*-**5** ($1\cdot10^{-5}$ M in MeOH) at 0 °C, -5 °C, -10 °C and -15 °C (Fig. 5b, 6b, 7b)

$$\frac{\ln k}{T} = -\frac{\Delta H^{\ddagger}}{R}\cdot\frac{1}{T} + \frac{\ln k_B}{h} + \frac{\Delta S^{\ddagger}}{R}$$

where k is reaction rate constant, T is absolute temperature, $\Delta H^{\ddagger}$ is the enthalpy of activation, R is gas constant, k_B is Boltzmann constant, h is Plank's constant and $\Delta S^{\ddagger}$ is the entropy of activation.

Clear isosbestic points were found at λ = 318 nm for the isomerization from stable *trans* to unstable *cis* form and at λ = 330 nm for the isomerization from unstable *cis* to stable *cis* form.

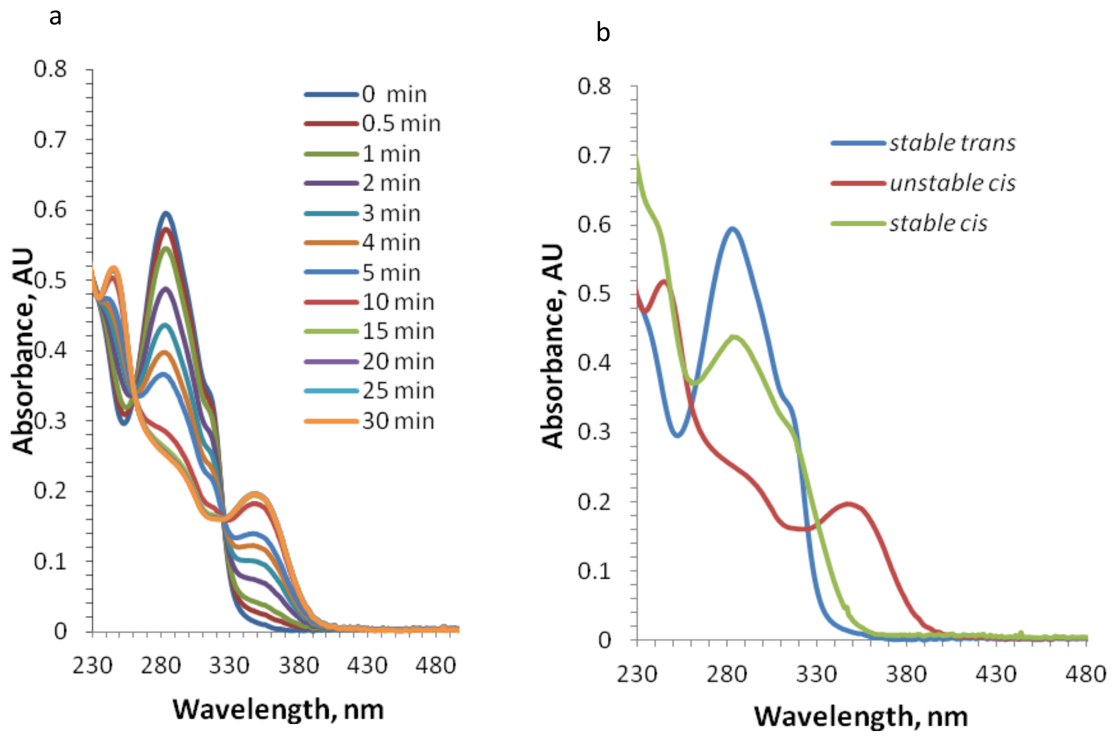

Figure S5. a) Changes in UV/vis spectrum upon irradiation at λ = 312 nm of compound *trans*-**5** ($1\cdot10^{-5}$ M in methanol). b) UV-vis Spectra of the three isomers stable at rt.

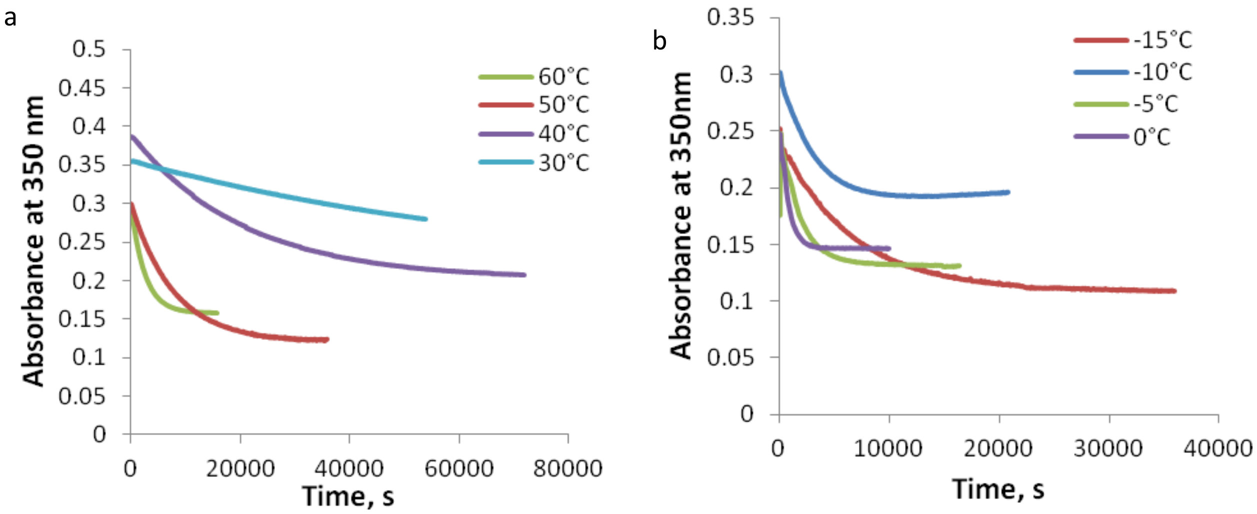

Figure S6. a) Exponential decrease of the absorption band at λ = 350 nm for unstable *cis*-**5** at different temperatures. b) Exponential decrease of the absorption band at λ = 350 nm for unstable *trans*-**5** at different temperatures.

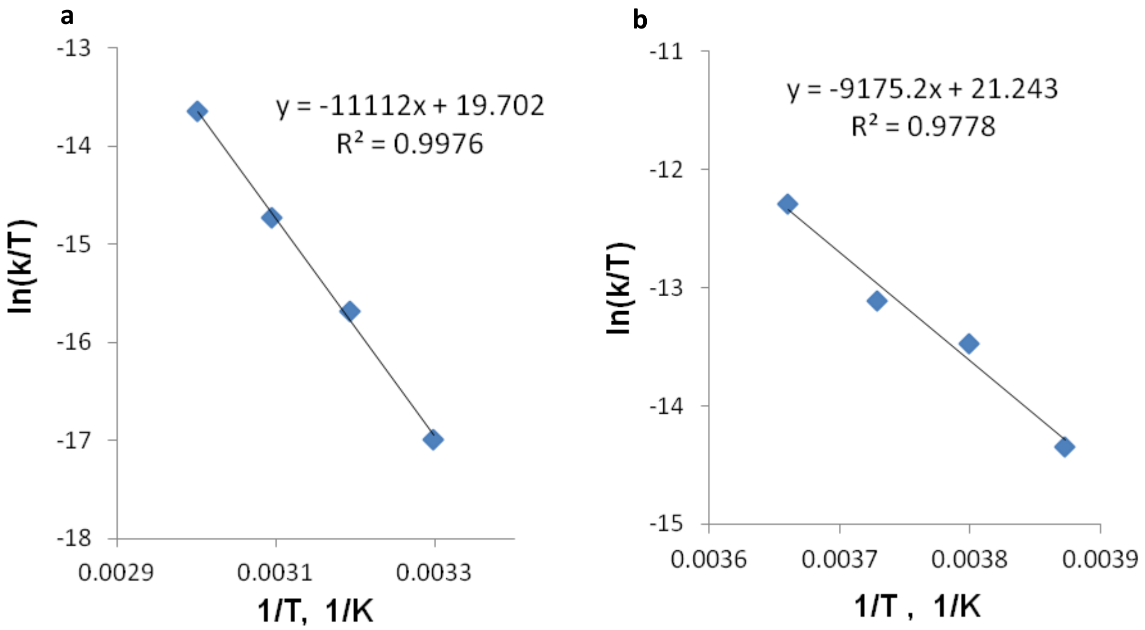

$\Delta^{‡}G°$= 102 kJ mol^{-1} $t_{1/2}$ (20 °C) = 2.2 d
$\Delta^{‡}H°$= 92 kJ mol^{-1}
$\Delta^{‡}S°$= –33 J mol^{-1}K^{-1}

$\Delta^{‡}G°$= 82 kJ mol^{-1} $t_{1/2}$ (20 °C)= 55 sec
$\Delta^{‡}H°$= 72 kJ mol^{-1}
$\Delta^{‡}S°$= –10 J mol^{-1}K^{-1}

Figure S7. a) Eyring plot for the thermal isomerization from unstable *cis*-**5** to stable *cis*-**5** and activation parameters. b) Eyring plot for the thermal isomerization from unstable *trans*-**5** to stable *trans*-**5** and activation parameters.

Isomerisation cycle of compound 1

UV-vis and NMR spectroscopy and HPLC were used to follow the isomerization steps of compound **1**. The cycle is shown in Fig. S8.

HPLC was used to determine the PSS (photostationary state) ratio's and the UV-vis spectroscopy to determine the activation parameters.

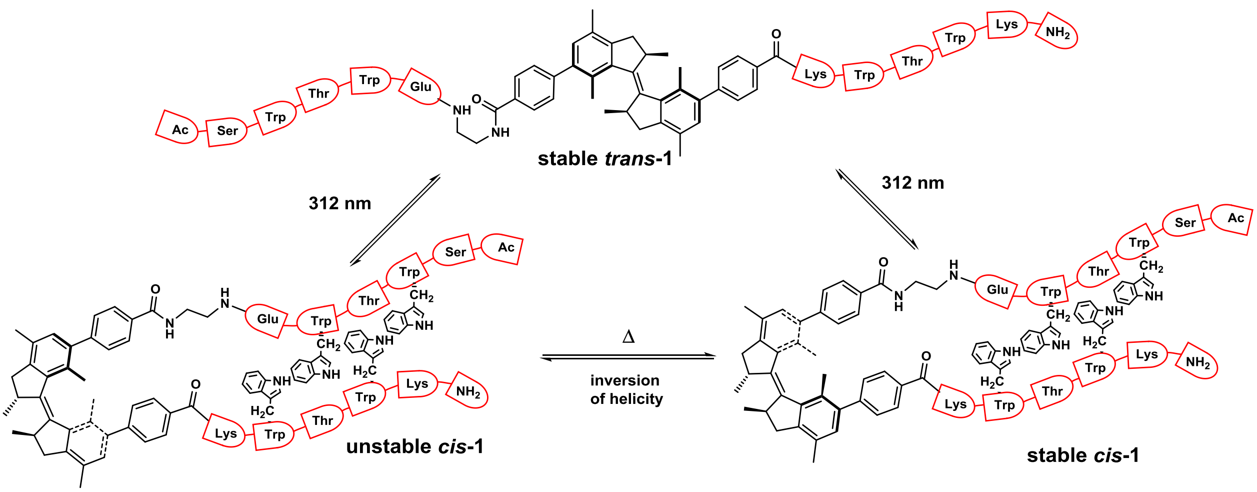

Figure S8. Isomerisation cycle for compound **1**.

Rotary cycle for compound 1 using HPLC analysis

A sample of compound *trans*-**1** (1 mg/mL) was prepared in 50:50 milliQ water:acetonitrile and analyzed by HPLC. The sample was irradiated for 2 min at λ = 312 nm, to obtain unstable *cis*-**1**, and analysed. Subsequently it was left for 4h at 60 °C, to obtain stable *cis*-**1**, followed by HPLC analysis. The HPLC traces are reported in Fig. S9.

a

R. time (trans) =32.56 min

Purity 96%

b

2 min irradiation at 312 nm

R. time (unstable cis) =38.38 min

PSS: 93% at 324 nm (isosbestic point)

c

4 h at 60 °C

R. time (stable cis) =38.35 min

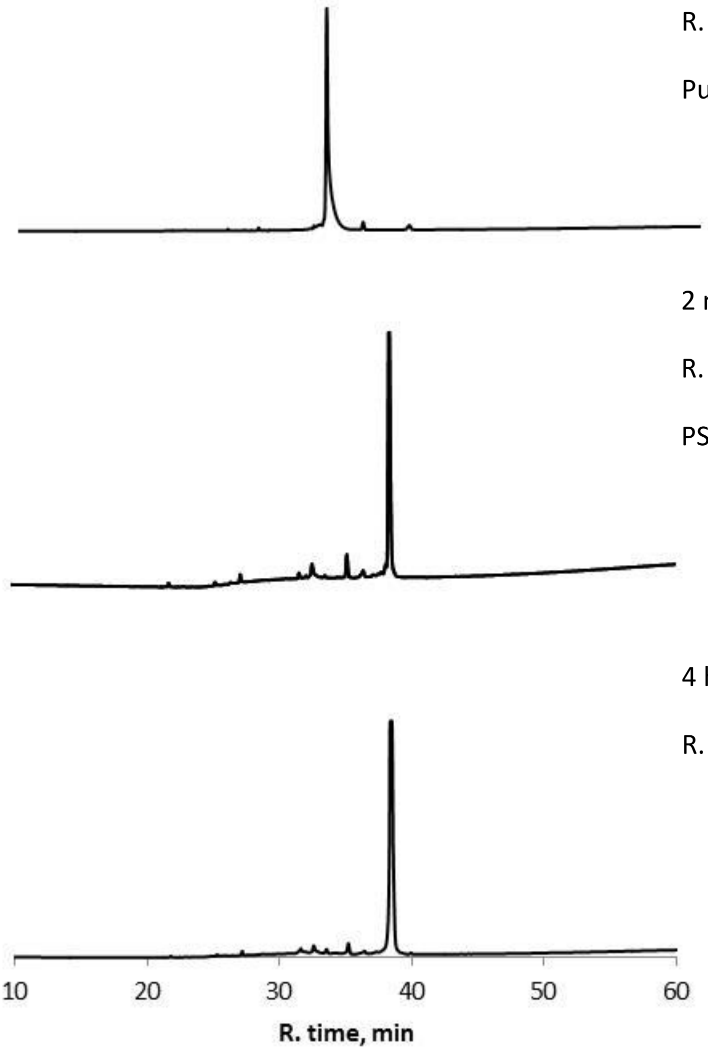

Figure S9. a) HPLC trace (detection wavelength: 220 nm) for *trans*-**1**. b) HPLC trace (detection wavelength: 220 nm) for unstable *cis*-**1**. c) HPLC trace (detection wavelength: 220 nm) for stable *cis*-**1**.

Kinetic study of compound 1 using UV-vis spectroscopy

The same procedure as reported for compound **5** was used. The temperature used are 40 °C, 45 °C, 50 °C, 55 °C and 60 °C.

a b

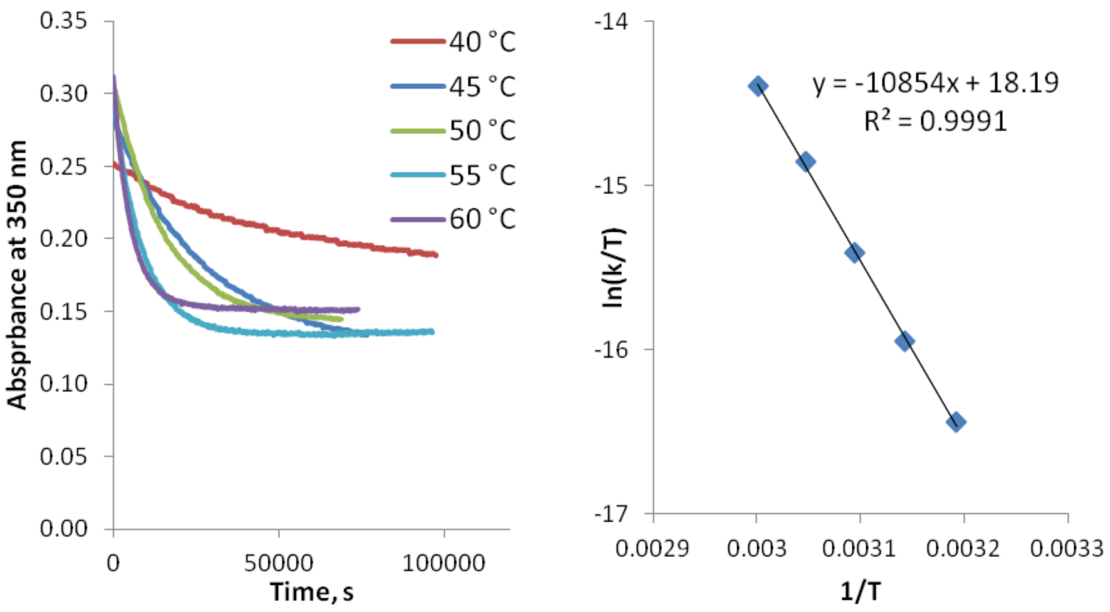

$\Delta^{\ddagger}G°$= 104 kJ mol^{-1} $t_{1/2}$ (20 °C) = 4.1 d

$\Delta^{\ddagger}H°$= 90 kJ mol^{-1}

$\Delta^{\ddagger}S°$= –46 J mol^{-1}K^{-1}

Figure S10. a) Exponential decrease of the absorption band at λ = 350 nm for unstable *cis*-**1** at different temperature. b) Eyring plot for the thermal isomerization from unstable *cis*-**1** to stable *cis*-**1** and activation parameters

CD Spectroscopy for compound 3

A 24 uM solution of stable *trans*-**3** in MeOH at 20 °C was irradiated at λ = 312 nm and then warmed at 40 °C. CD spectra were recorded before and after irradiation at λ = 312 nm and after warming. For each isomer, CD spectra were recorded at 5 °C and 20 °C. Spectra are shown in Fig. S11.

a

b

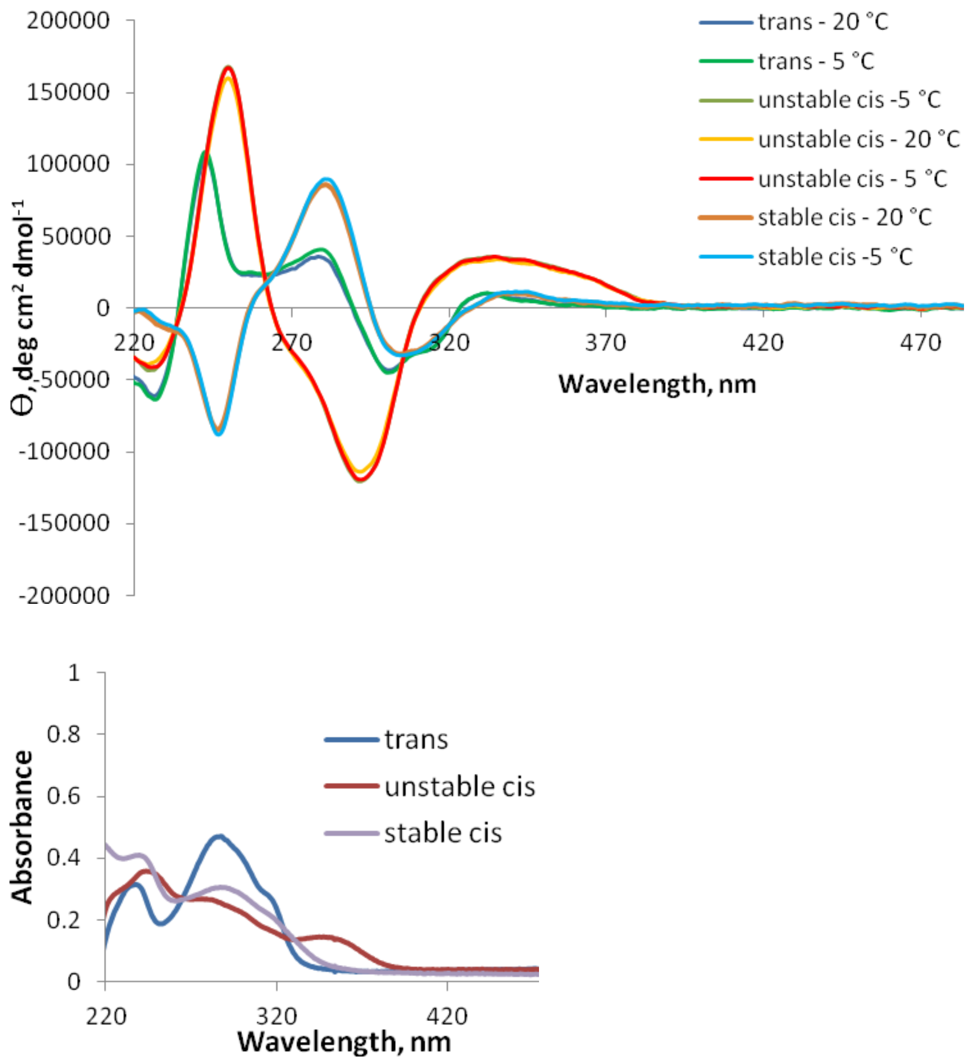

Figure S11. a) CD spectra of *trans*-**3**, unstable *cis*-**3** and stable *cis*-**3** at 5 °C and 20 °C. b) UV-Vis spectra of *trans*-**3**, unstable *cis*-**3** and stable *cis*-**3**.

CD Spectroscopy for compound 1

A 24 uM solution of stable *trans*-**1** in MeOH at 20 °C was irradiated at λ = 312 nm and then warmed at 40 °C. CD spectra were recorded before and after irradiation at λ = 312 nm and after warming. For each isomer, CD spectra were recorded at different temperature, between -10 °C and 40 °C. Spectra are shown in Fig. S12.

a

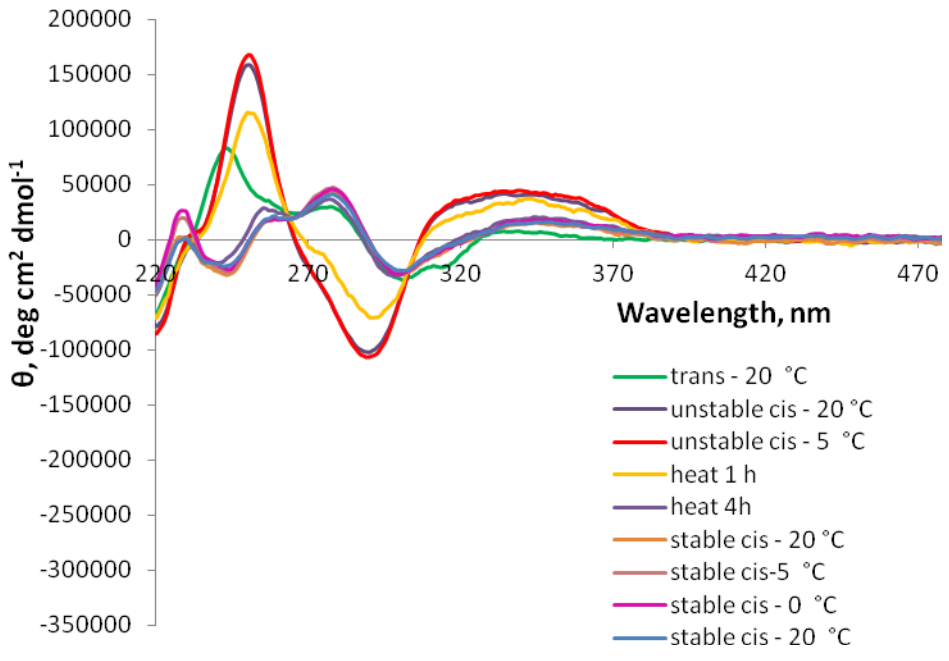

b

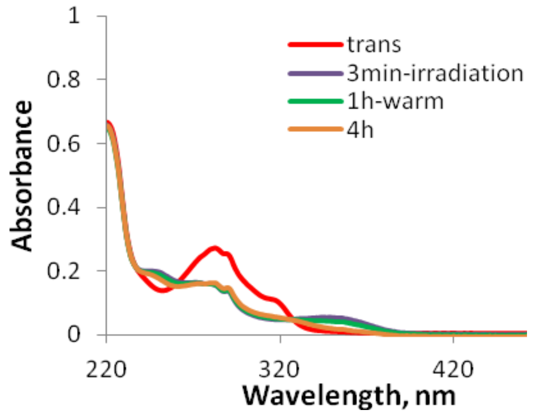

Figure S12. a) CD spectra of *trans*-**1**, unstable *cis*-**1** and stable *cis*-**1** at 5 °C and 20 °C. b) UV-Vis spectra of *trans*-**1**, unstable *cis*-**1** and stable *cis*-**1**.

Temperature dependency for CD signal at 228 nm for compound 1

A 24 uM solution of stable *trans*-**1** in MeOH was prepared. The solution was irradiated at λ = 312 nm and then warmed to 40 °C. CD spectra was recorded before and after irradiation at λ = 312 nm and after warming. For every isomer, CD spectra were recorded from 0 °C till 40 °C with intervals of 5 °C. Spectra are shown in Fig. S13.

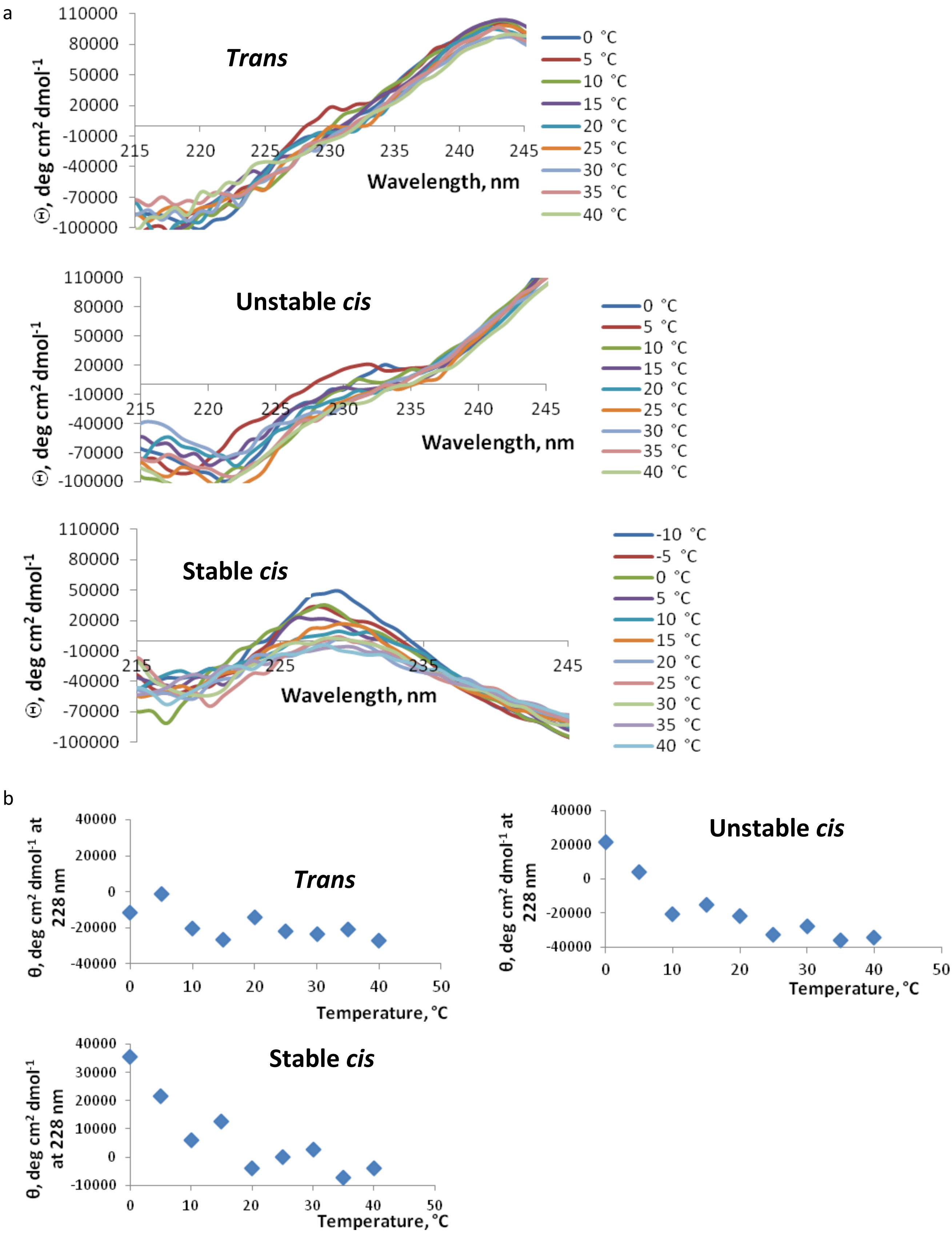

Figure S13. a) CD spectra (215-245 nm region) of *trans*-**1**, unstable *cis*-**1** and stable *cis*-**1** at different temperatures. b) Molar ellipticity at 228 nm for the three isomers measured at different temperatures.

CD Spectroscopy for compound 8

A 71 uM solution of **8** in MeOH was prepared. CD spectra were recorded from -10 °C till 40°C with interval of 5 °C. Spectra are shown in Fig. S14.

a

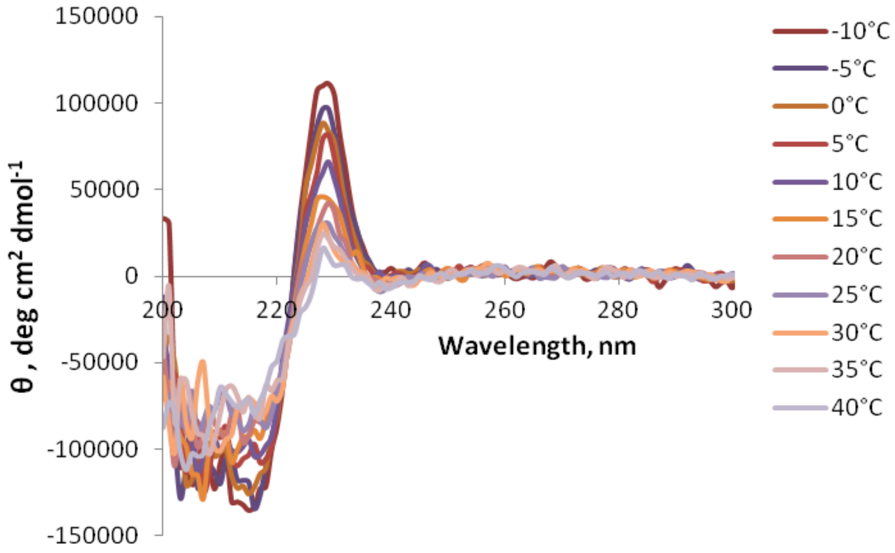

b

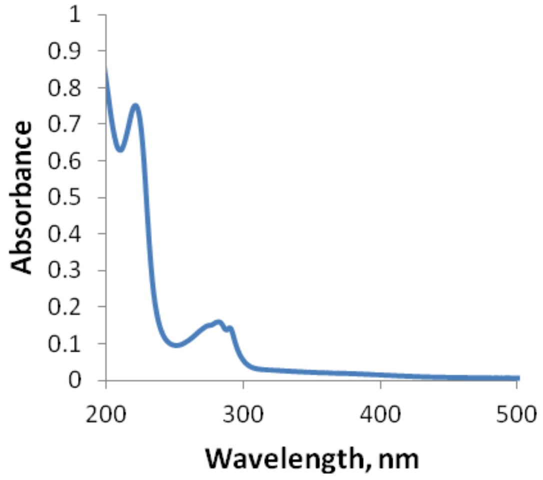

c

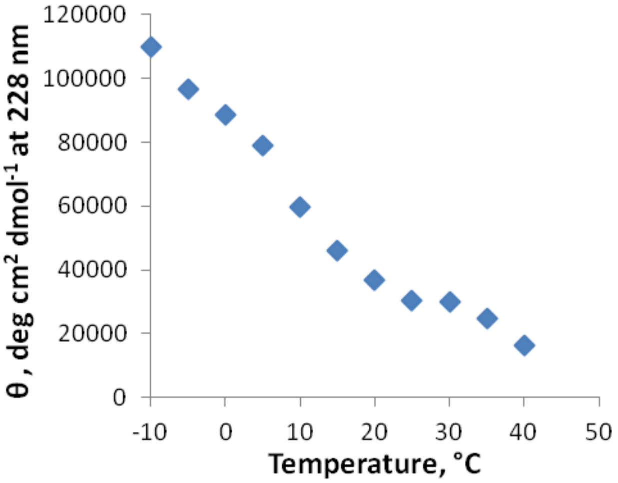

Figure S14. a) CD spectra of **8** at different temperatures. b) UV-vis spectrum of **8**. c) Molar ellipticity of **8** at 228 nm at different temperatures.

2D-NMR Studies on compound 1

A solution of compound **1** (1.8 mg in 300 uL methanol-d3) was used.

***Trans*-trpzipper-overcrowded alkene**

AA	NH	Alpha-H	Others
Ac			
Ser-1	8.07	4.09	3.65, 3.57 β-H
Trp-2	7.87	4.57	3.20 β-H
Thr-3	7.54	3.97	3.96 β-H, 0.73 γ-H
Trp-4	7.61	4.57	
Glu-5	7.78	4.20	1.88, 2.04 β-H, 2.18 γ-H
Switch	8.40(Mot),<br>7,58 (Glu)		3.55, 3.50 Switch-NH-C**H**$_2$, 3.42 Glu-NH-C**H**$_2$<br>6.86 arom singlet (Glu), 6.93 arom singlet (Lys)<br>7.79 (ortho), 7.47 (meta) Lys<br>7.91 (ortho), 7.43 (meta) Glu<br>2.22 arom CH$_3$ (Glu), 2.27 arom CH$_3$ (Lys)<br>1.07,1.11 CHC**H**$_3$<br>3.02, C**H**CH$_3$<br>2.35, 2.72 C**H**$_2$CHCH$_3$
Lys-6	8.55	4.34	1.76, 1.57, 1.28 (δ), 2.80- 2.02(ε?)
Trp-7	7.99	4.70	
Thr-8	7.61	4.23	4.13 β-H, 0.94 γ-H
Trp-9	7.97	4.57	
Lys-10	7.87	4.22	1.77,1.53, 1.30 (δ), 2,85-2.07(ε?)
NH2			

Aromatic protons for Trp

A	B	C	D	E	F
7.60	7.03	7.10	7.32	7.20	10.34
7.56	7.97	7.06	7.30	7.12	10.37
7.54	6.95	7.03	7.29	7.12	10.28
7.49	6.96	7.06	7.3	7.18	10.27

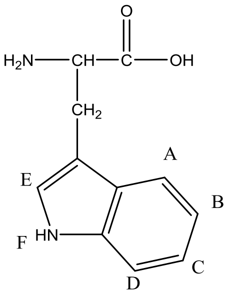

NOE interactions

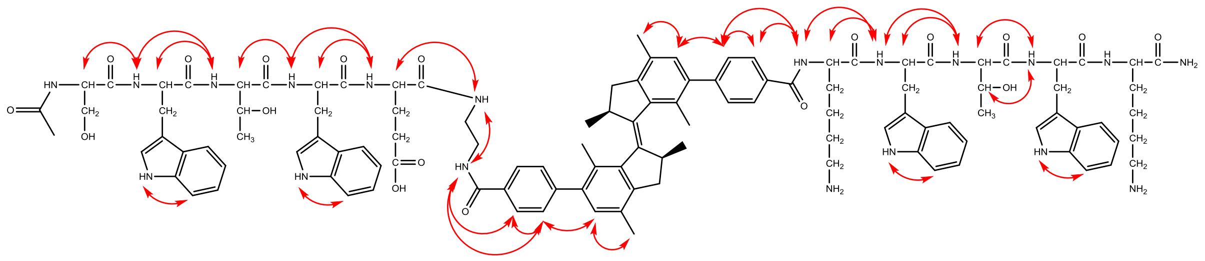

Stable *cis*-trpzipper-overcrowded alkene

**AA**	**NH**	**Alpha-H**	**Others**
Ac			2.18
Ser-1	8.11	4.07	3.68, 3.59 β-H
Trp-2			β-H
Thr-3	7.59	3.92	3.89 β-H, 0.69 γ-H
Trp-4			
Glu-5	8.06	4.20	2.20 β-H γ-H
Switch	8.41 (Mot),<br>7.75 (Glu)		3.38 SwitchNH-C**H**$_2$, 3.65 Glu-NH-C**H**$_2$<br>6.85 arom singlet (Glu), 6.77 arom singlet (Lys)<br>7.36 (ortho), 7.92 (meta) Lys<br>7.37 (ortho), 7.95 (meta) Glu<br>2.26 arom CH_3 (Glu) external, 1.45 internal<br>2.24 arom CH_3 (Lys) external, 1.37 internal<br>1.13 CHC**H**$_3$<br>3.45 C**H**CH_3<br>2.56, 3.12 C**H**$_2$$CHCH_3$
Lys-6	8.68	4.26	1.67, 1.56 (δ or γ or β), 2.77 (ε)
Trp-7	8.13	4.73	3.28, 3.16
Thr-8	7.77	4.22	4.09 β-H, 0.92 γ-H
Trp-9	8.06	4.56	3.19
Lys-10	7.90	4.23	1.27, 1.55, 1.78 (δ or γ or β),3.21 (ε)
NH2			

Trp:

**NH**	**Alpha-H**	**Others**
7.95	4.54	3.15
7.61	4.53	3.08, 3.15 β-H

Aromatic protons for Trp :

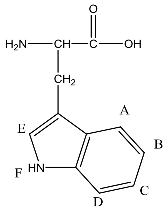

**A**	**B**	**C**	**D**	**E**	**F**
7.45	6.87	6.93	7.19	6.94	10.14
7.53	6.99	7.07	7.30	7.14	10.29
7.21	6.95	6.80	6.88	7.08	10.34
7.42	6.88	7.02	7.27	7.07	10.09

In yellow: The A, B, C, D system has not been correlated with E, F system.

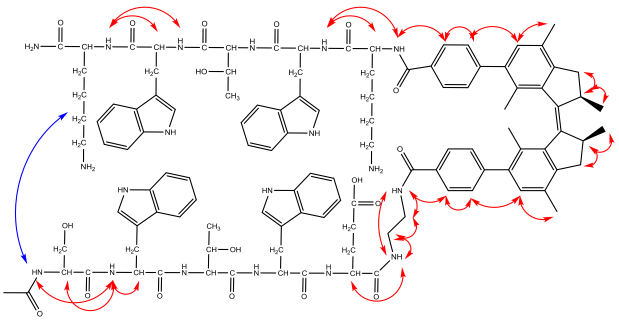

Stable *cis*-trpzipper-overcrowded alkene (5 °C)

AA	NH	Alpha-H	Others
Ac			2.18
Ser-1	8.26	4.09	3.68, 3.58 β-H
Trp-2	8.11	4.55	3.28, 3.15 β-H
Thr-3	7.68		3.89 β-H, 0.66 γ-H
Trp-4	7.68		
Glu-5	7.8	4.13	1.89, 2.03 β-H, 2.14 γ-H
Switch	8.50 (Mot), 7.80 (Glu)		3.36 SwitchNH-C**H**$_2$, 3.64 Glu-NH-C**H**$_2$<br>6.86 Arom singlet (Glu), 6.80 Arom singlet (Lys)<br>7.37 (ortho), 7.94 (meta) Lys<br>7.38 (ortho), 7.96 (meta) Glu<br>2.26 arom CH_3 (Glu) external, 1.43 internal<br>2.24 arom CH_3 (Lys) external, 1.36 internal<br>1.08, 1.13 CHC**H**$_3$<br>3.46 C**H**CH_3<br>2.55, 3.12 C**H**$_2$CHCH_3
Lys-6	8.74	4.20	1.63, 1.53 (δ or γ or β)
Trp-7	8.22	4.75	3.29, 3.14
Thr-8	7.68	4.14	4.09 β-H, 0.93 γ-H
Trp-9	8.19	4.56	3.26, 3.18
Lys-10	8.04	4.23	1.24, 1.52, 1.80 (δ or γ or β)
NH2			

Aromatic protons for Trp :

Trp	A	B	C	D	E	F
2	7.40	7.00	6.85	7.26	7.10	10.44
4	7.29	6.95	6.79	7.20	6.94	10.25
7	7.46	6.94	6.86	7.19	7.06	10.18
9	7.51	7.06	6.99	7.30	7.14	10.38

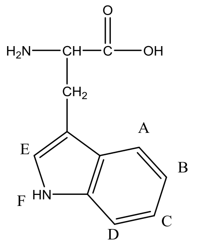

In same colors: Protons that have a correlation in NOESY.

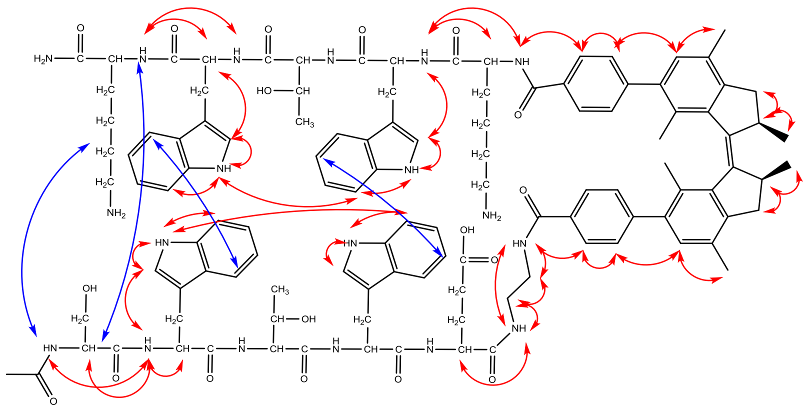

Irradiation at 312 nm

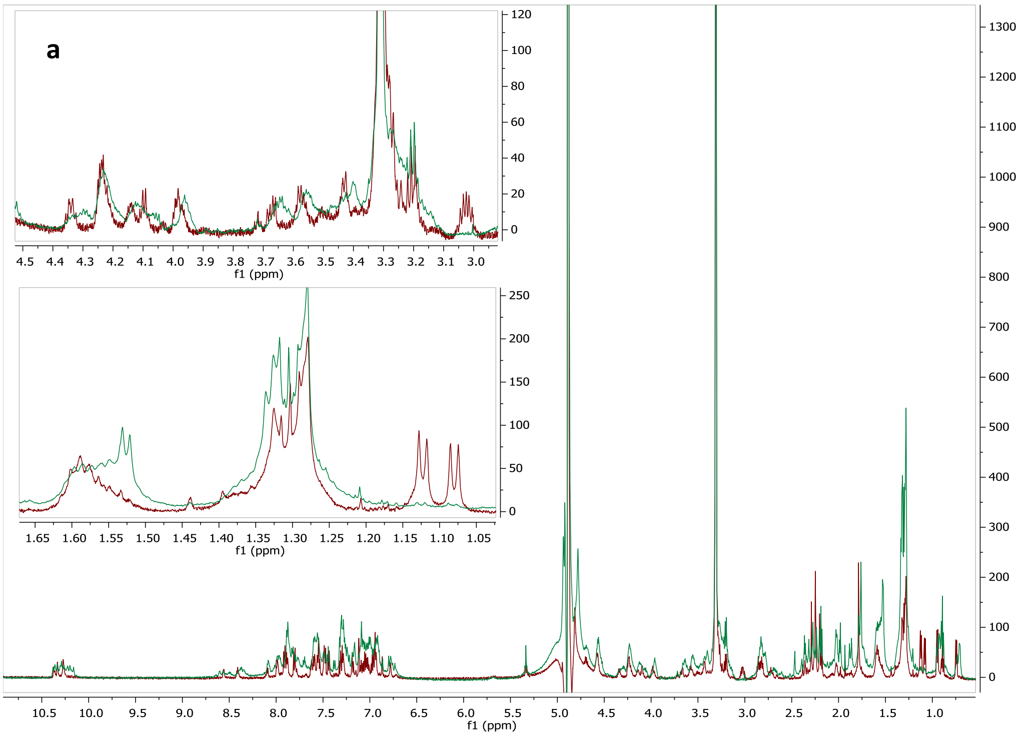

Warming up

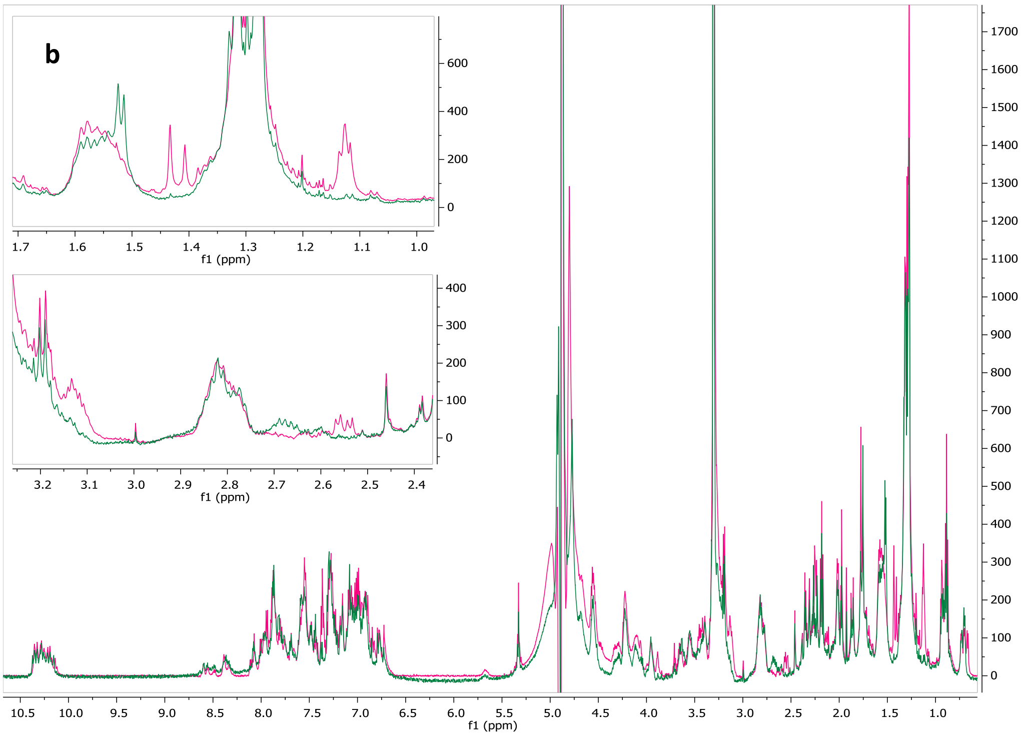

Figure S15. Rotary cycle of compound **1** (1.8 mg in 300 uL methanol-d3). a) NMR spectra before (red line) and after (green line) irradiation at λ= 312 nm for 30 min. b) NMR spectra before (green line) and after (pink line) warming up at 60 °C for 4 h. The expanded regions (inset) correspond to the CH_3 in the connected to the aromatic system.

Stable *cis*-overcrowded alkene-β hairpin (at rt).

^{1}H-NMR.

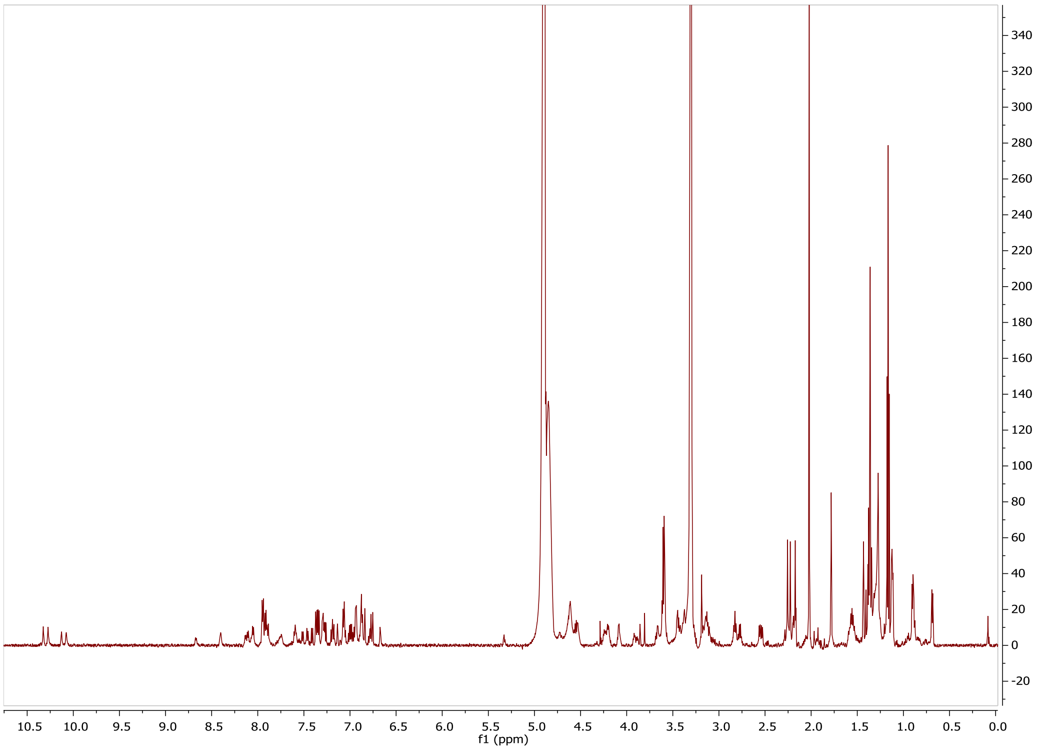

Stable *cis*-overcrowded alkene-β hairpin (at 5 °C).

^{1}H-NMR.

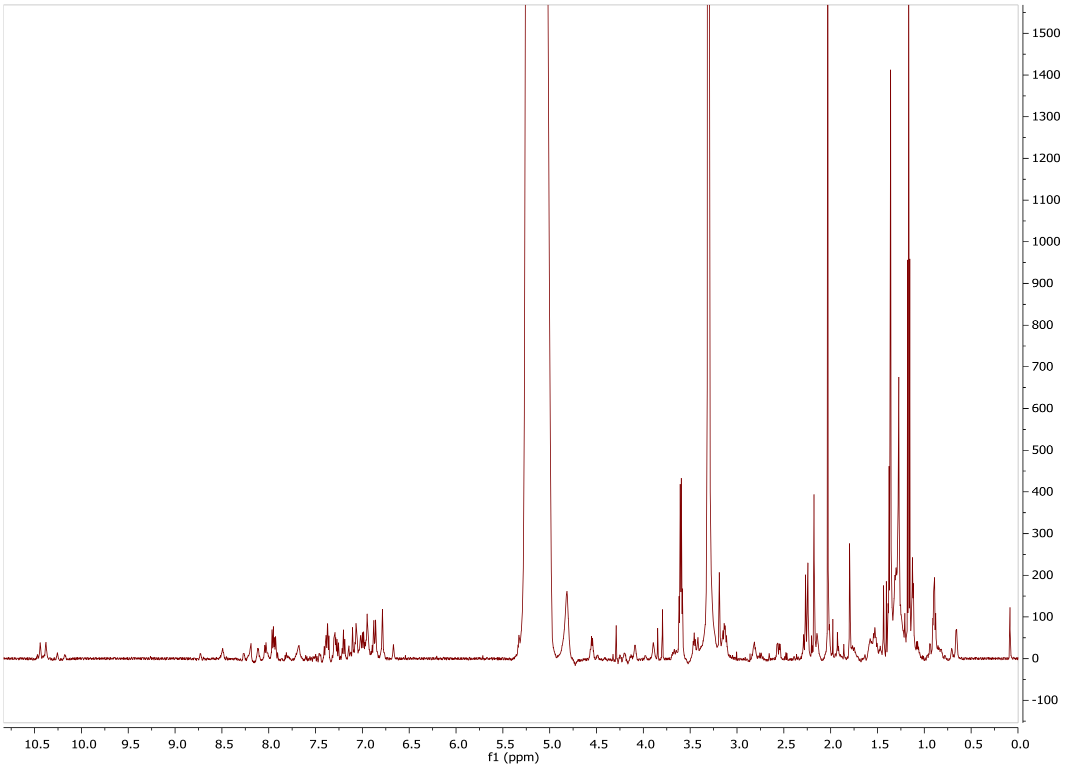

NOESY NH region.

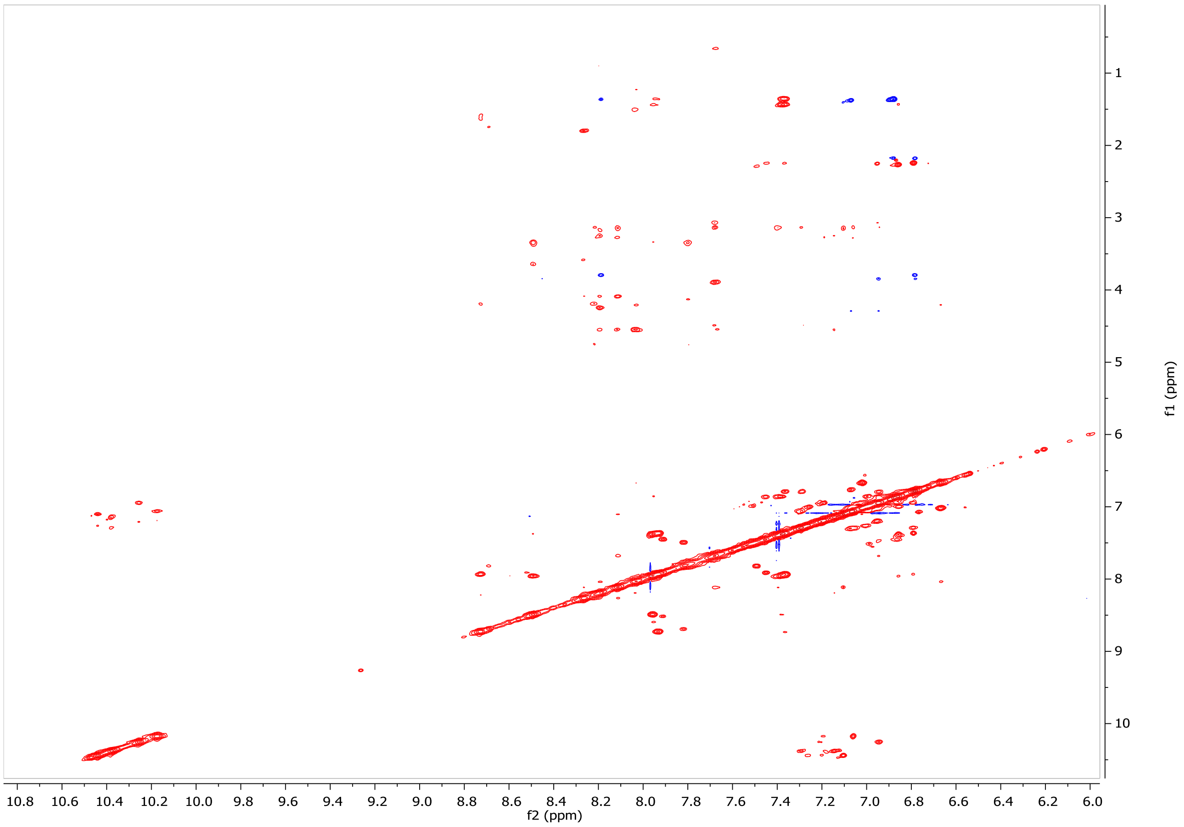

TOCSY NH region.

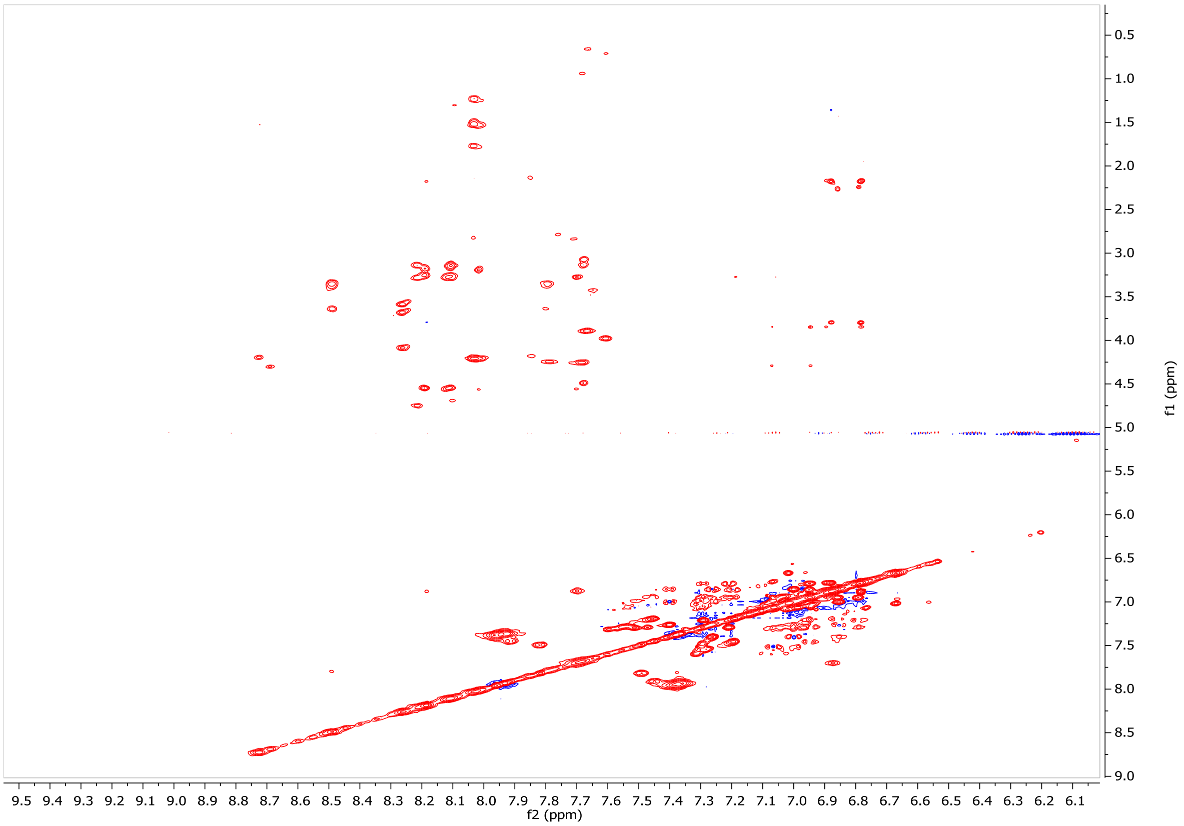

Stable *trans*-overcrowded alkene-β hairpin.

^{1}H-NMR

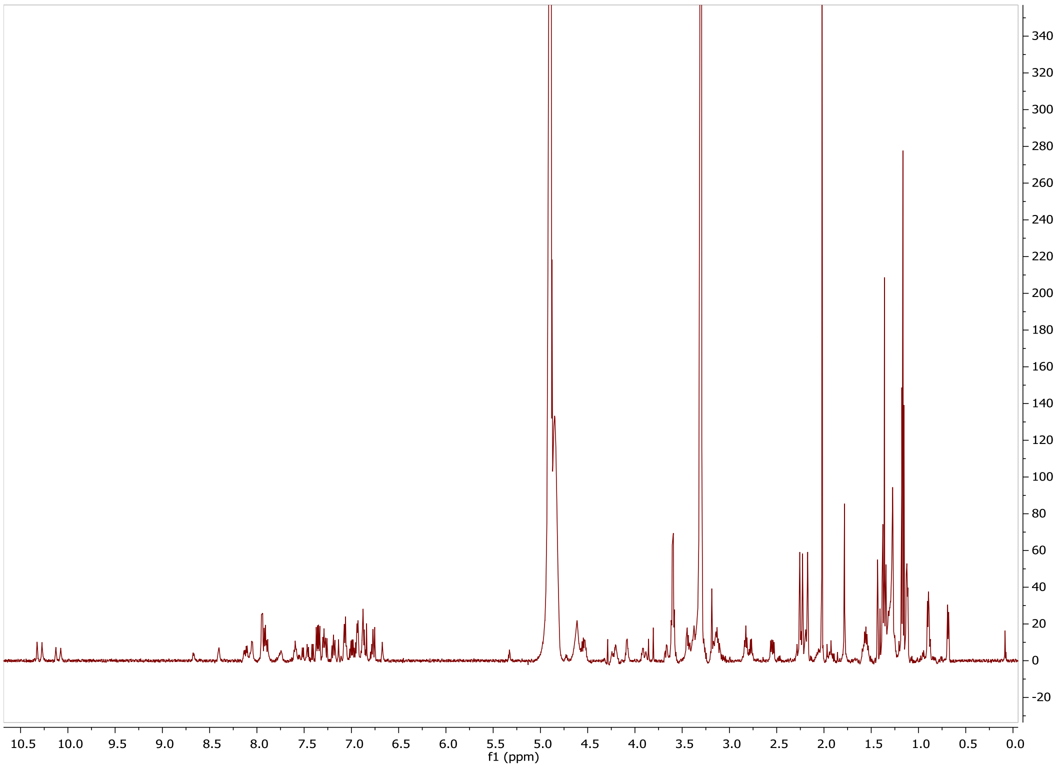

NH region, TOCSY.

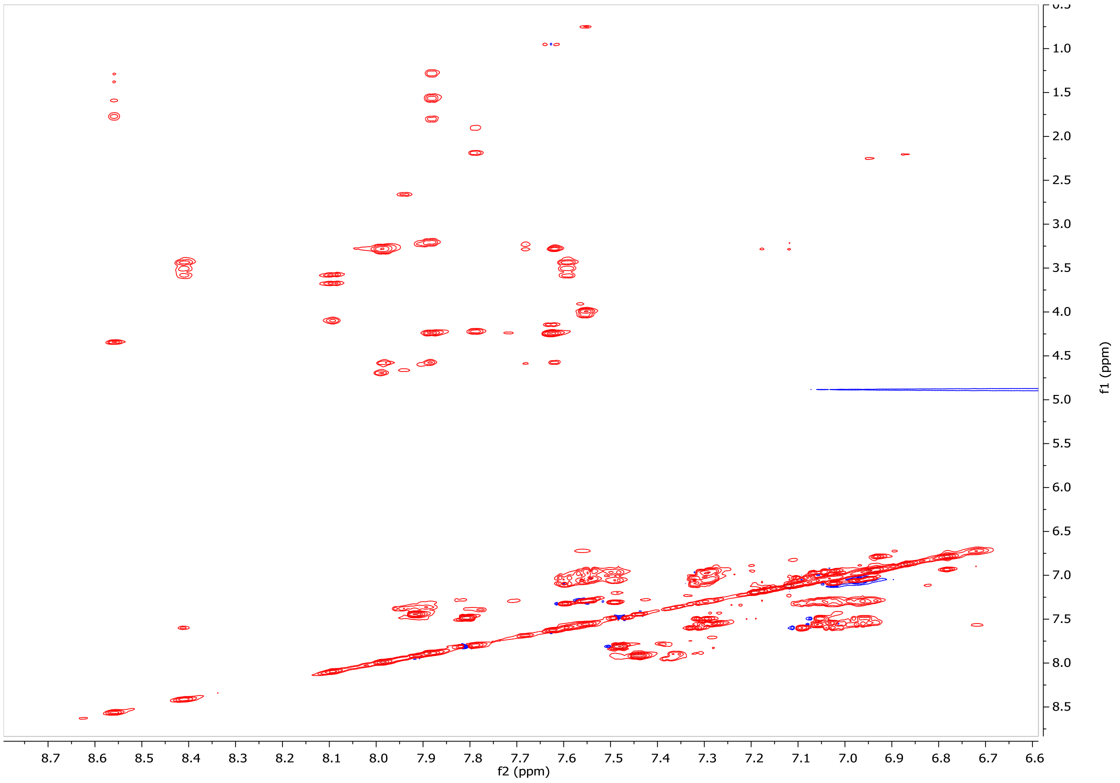

NH region, NOESY.

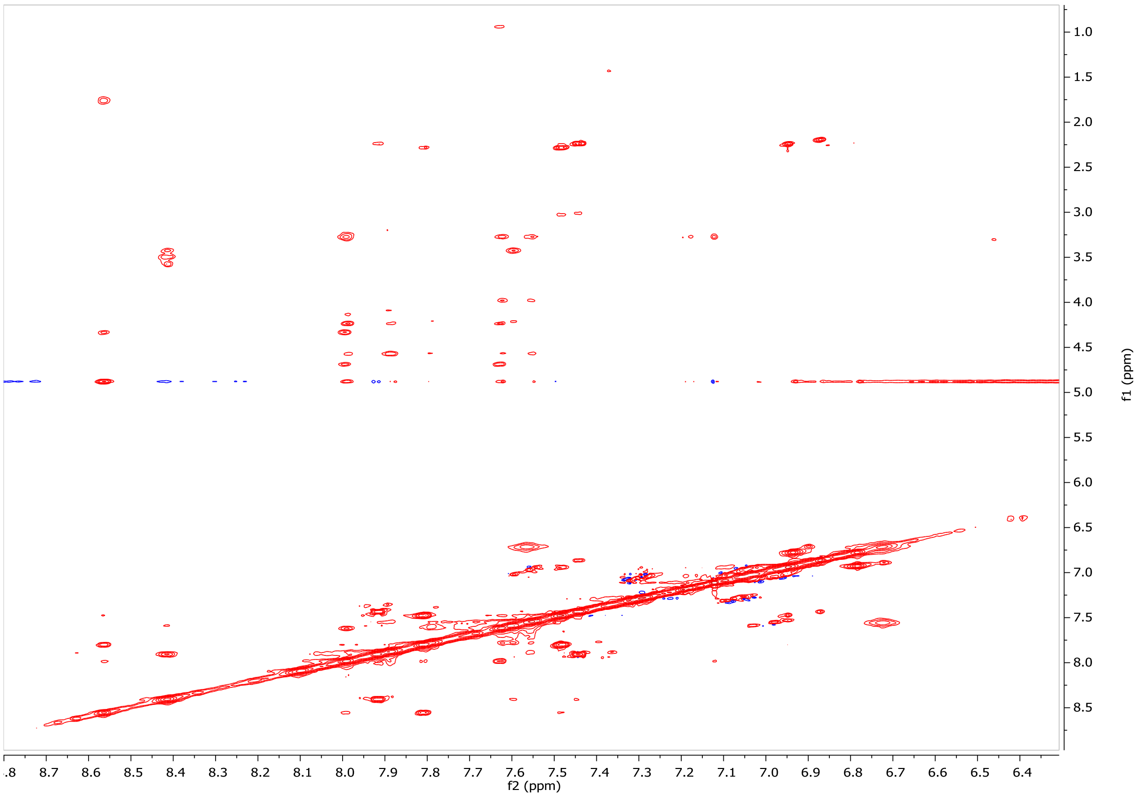

TEM measurements for compound 1

Preparation of TEM-samples. Samples for transmission electron microscopy were placed on carbon coated 400 mesh copper grids. After 1 min of adhesion the sample was removed by filter paper and stained with 2% uranyl acetate and dried with filter paper. The samples were measured in a Philips CM120 or FEI T20 electron microscope operating at 120 or 200 keV.

Solutions of compound *trans*-**1** and stable *cis*-**1** (1.8 mg in 300 uL methanol-d3) were analyzed.

Cryo-TEM measurements for compound 1

Preparation of cryo TEM-samples. 3 µl sample was placed on a glow discharged holy carbon coated grid (Quantifoil 3.5/1) and blotted. Samples were subsequently vitrified in liquid ethane (FEI, Vitrobot). The samples were observed in a Philips CM120 or FEI T20 electron microscope operating at 120 or 200 keV using a Gatan cryo-stage. Images were recorded under low-dose conditions using a slow-scan CCD camera.

First procedure: A solution of *trans*-**1** in milliQ water (1 mg/mL) was prepared. The sample was irradiated for 5 min at λ= 312 nm to form unstable cis and then warmed up to 45 °C for 5 h. Cryo TEM images were taken before and after irradiation and after warming. Before taking the TEM images, a UV-vis sample was prepared to examine the composition of the system: 2 uL of the aqueous solution were taken and diluted with 200 uL of a different solvent (acetonitrile or methanol) (Fig. S16).

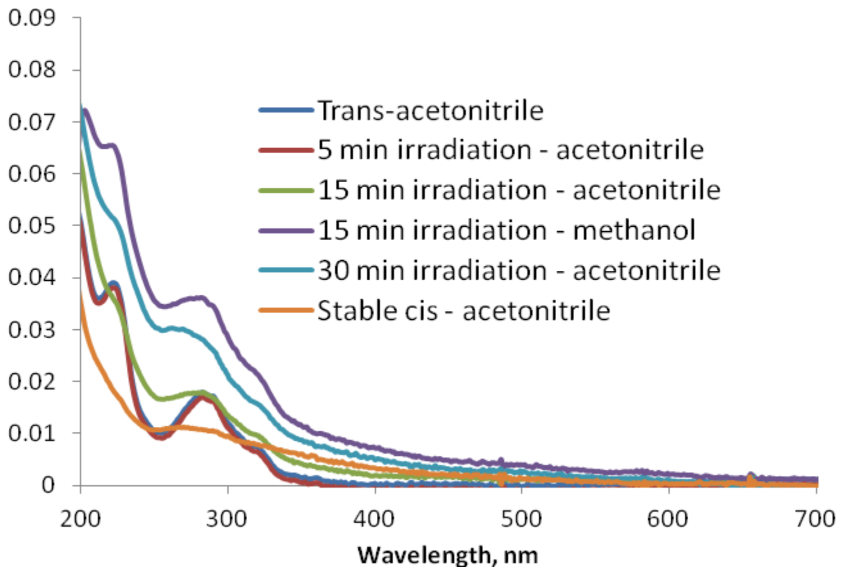

Figure S16. UV-vis spectra of *trans*-**1** at indicated stages during the irradiation process.

Trans (first procedure)

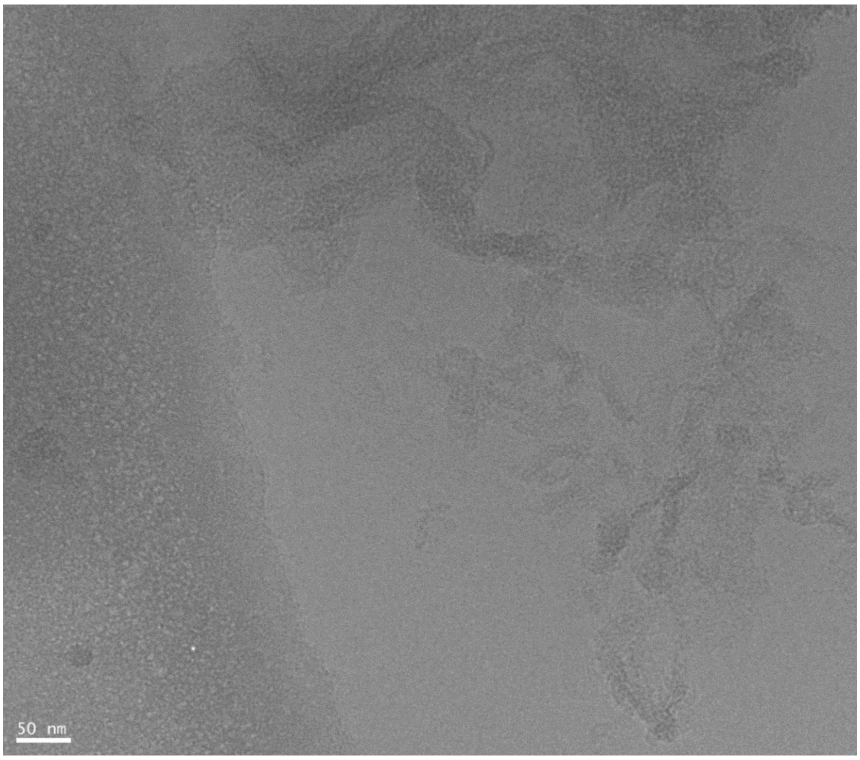

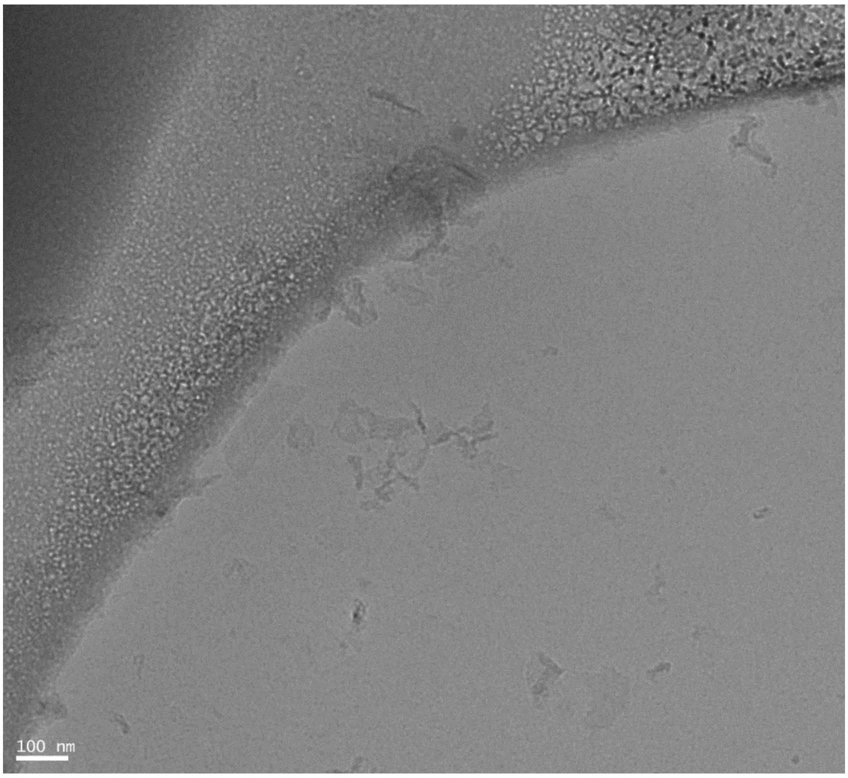

Unstable *Cis* (first procedure)

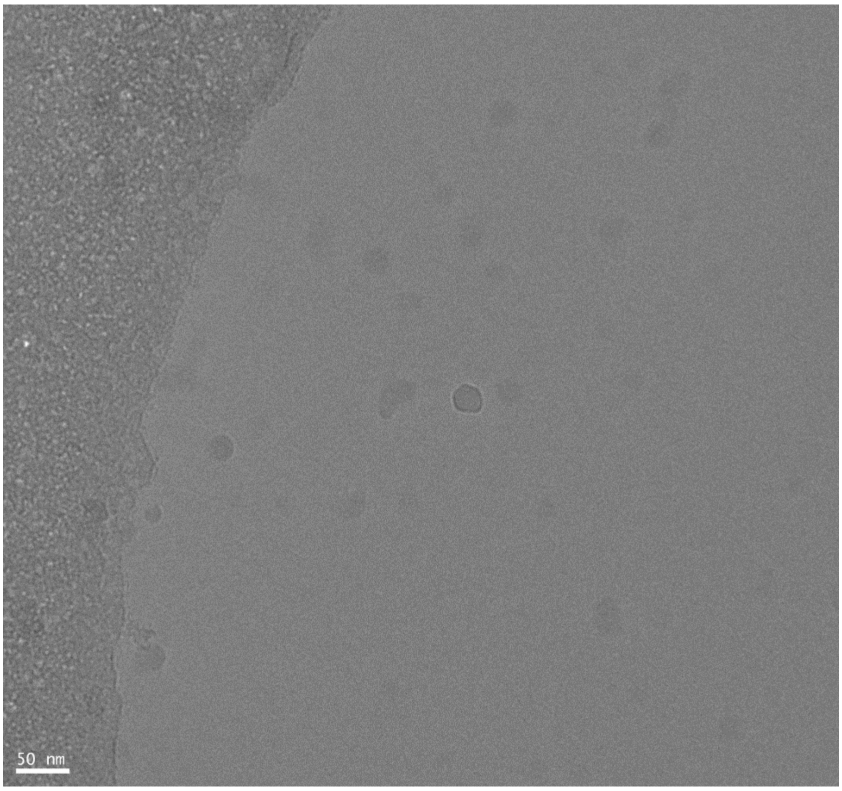

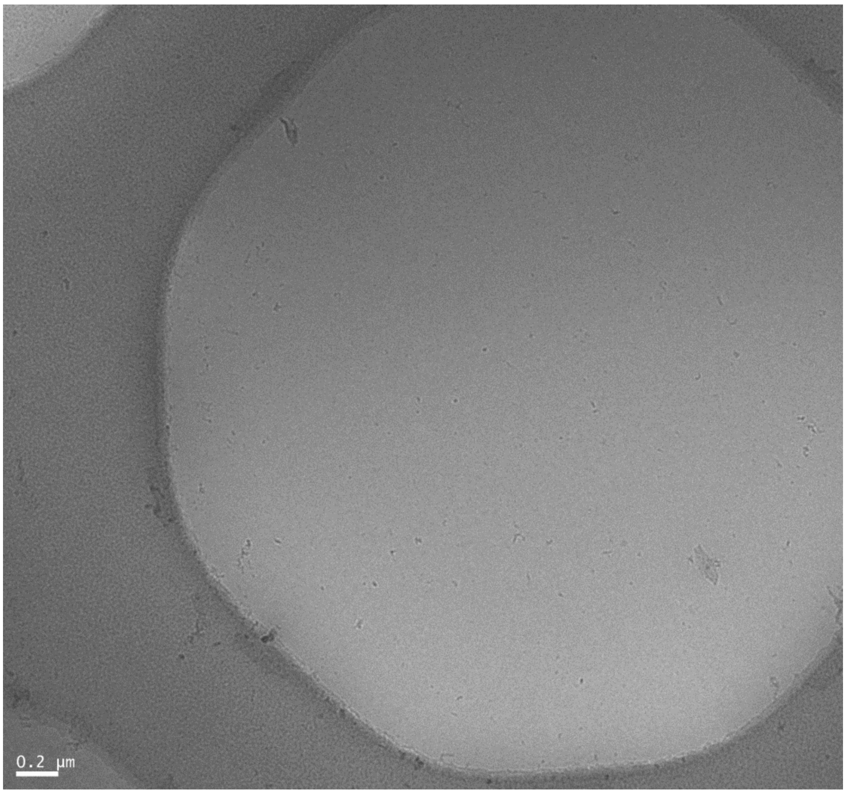

Stable *Cis* (first procedure)

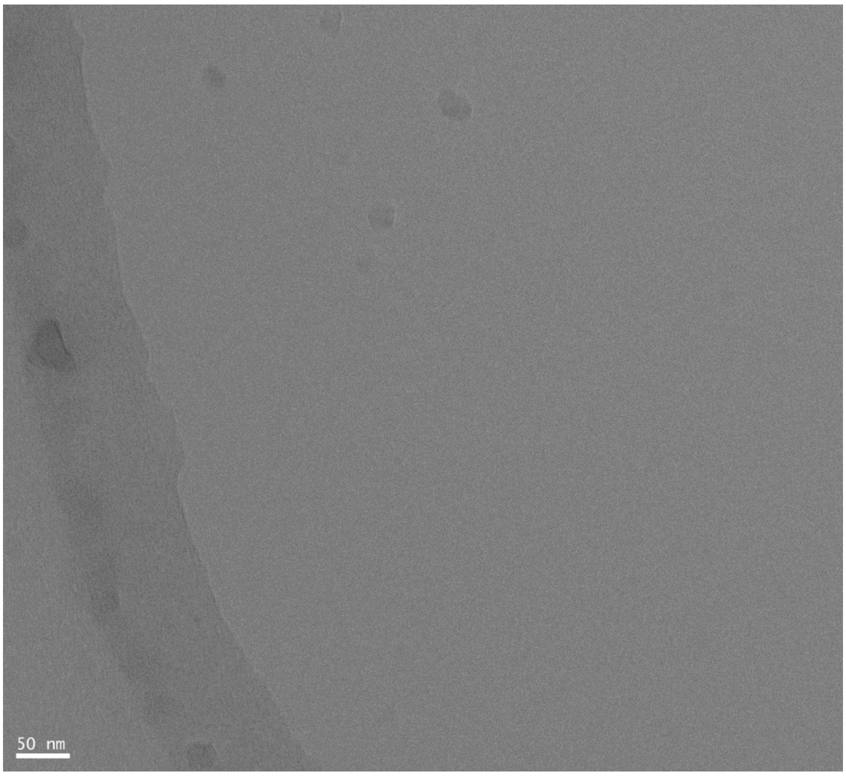

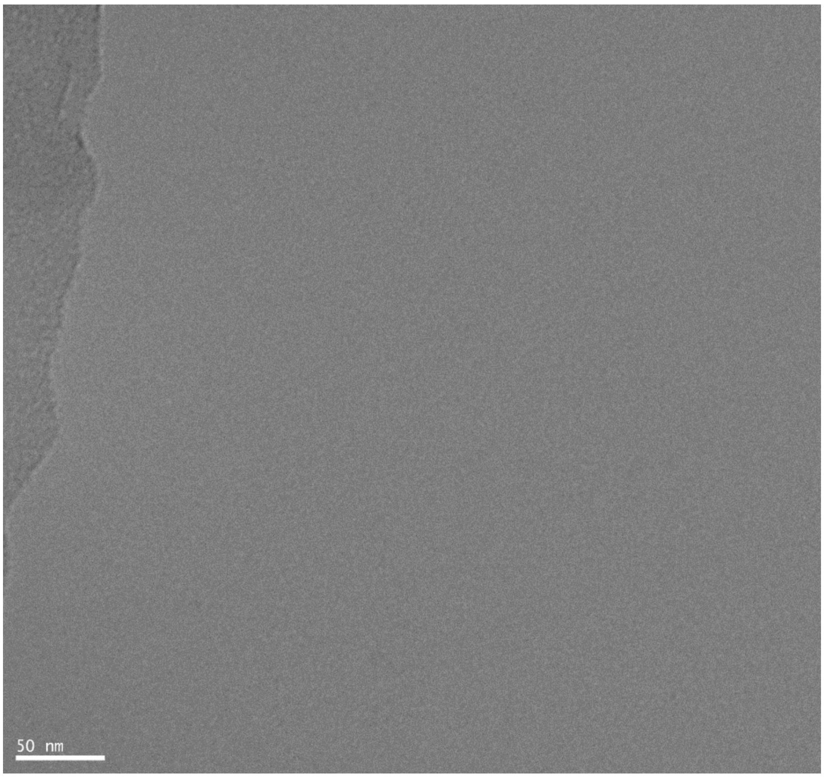

Second procedure: A second sample was prepared in MeOH (0.1 mg in 2 mL), irradiated for 5 min at λ= 312 nm to form unstable *cis*-**1**, the solvent was evaporated and 50 uL of milliQ water were added. 1 mL was then warmed up to 45 °C for 5 h, the solvent was evaporated and 50 uL of milliQ water was added (Fig. S17).

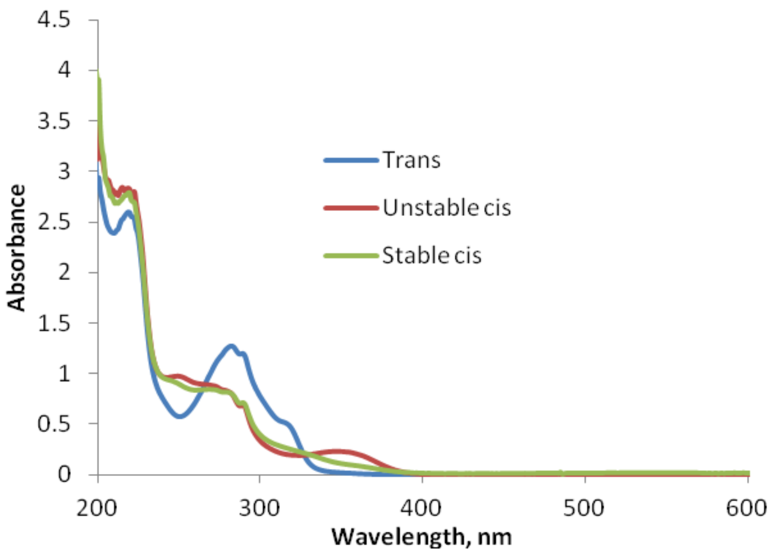

Figure S17. UV-vis spectra of *trans*-**1**, *unstable cis*-**1,** *stable cis*-**1** in methanol.

Unstable *Cis* (Second Procedure)

Stable *Cis* (Second Procedure)

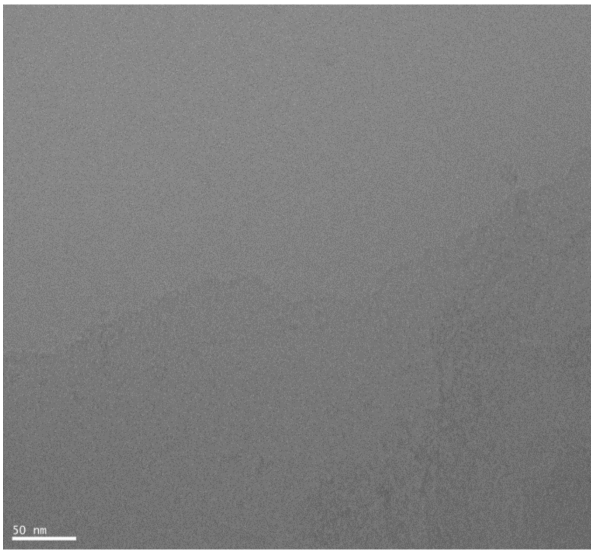

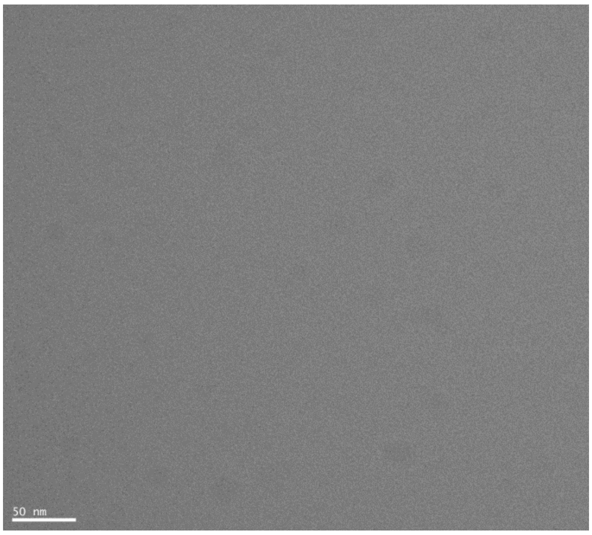

Nile red experiment

A sample of *trans*-**1** (1 mg/mL) was prepared in milliQ water. 250 uL of this solution were irradiated at λ= 312 nm for 30 min to obtain unstable *cis*-**1**. A sample of stable *cis*-**1** (1mg/mL) was prepared in milliQ water. 2uL of a solution of Nile red (0.25 mM in ethanol) was added to each of the three samples of *trans*-**1**, unstable *cis*-**1** and stable *cis*-**1**. Fluorescence emission spectra of Nile red were recorded at different excitation wavelength (500 nm, 525 nm, 550 nm). The fitting of the emission spectra are shown in Fig. S18.

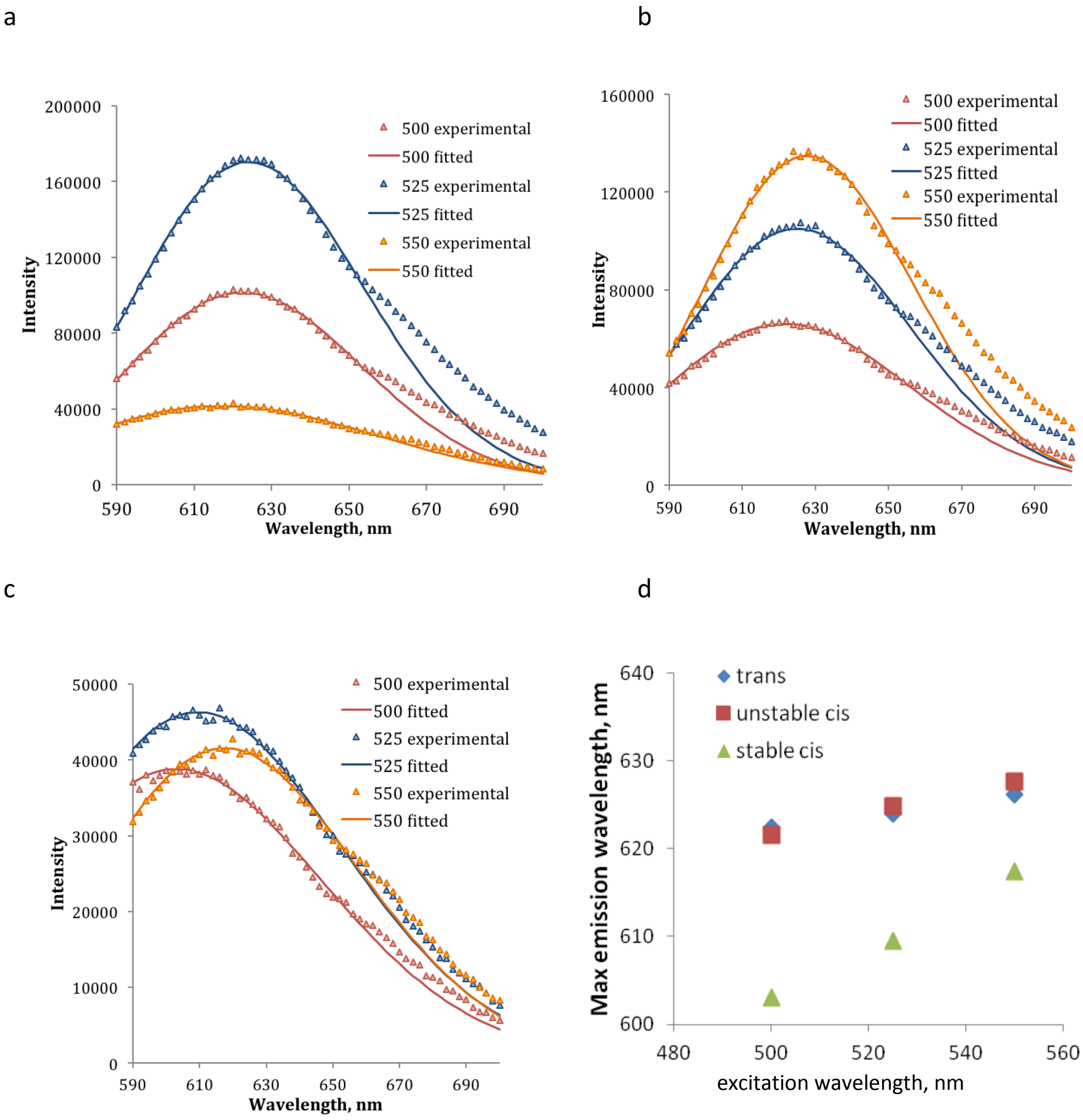

Figure S18. (a) Experimental and calculated emission spectra for *trans*-**1.** (b) Experimental and calculated emission spectra for unstable *cis*-**1**. (c) Experimental and fitted emission spectra for stable *cis*-**1**. (d) Plot of the excitation wavelengths versus the emission wavelengths maxima.
